# Hexokinase HK3-mediated O-GlcNAcylation of EP300: a key regulator of PD-L1 expression and immune evasion in ccRCC

**DOI:** 10.1038/s41419-024-06921-1

**Published:** 2024-08-23

**Authors:** Wei Zhang, Enyang Zhao, Zhuolun Li, Weiyang Liu, Jinpeng Wang, Wenbin Hou, Nan Zhang, Yang Yu, Xuedong Li, Bosen You

**Affiliations:** 1 Department of Urology, The Second Affiliated Hospital of Harbin Medical University, Harbin, China; 2 Future Medical Laboratory, The Second Affiliated Hospital of Harbin Medical University, Harbin, China

**Keywords:** Cancer metabolism, Renal cell carcinoma

## Abstract

Clear cell renal cell carcinoma (ccRCC) demonstrates enhanced glycolysis, critically contributing to tumor development. Programmed death-ligand 1 (PD-L1) aids tumor cells in evading T-cell-mediated immune surveillance. Yet, the specific mechanism by which glycolysis influences PD-L1 expression in ccRCC is not fully understood. Our research identified that the glycolysis-related gene (GRG) HK3 has a unique correlation with PD-L1 expression. HK3 has been identified as a key regulator of O-GlcNAcylation in ccRCC. O-GlcNAcylation exists on the serine 900 (Ser900) site of EP300 and can enhance its stability and oncogenic activity by preventing ubiquitination. Stably expressed EP300 works together with TFAP2A as a co-transcription factor to promote PD-L1 transcription and as an acetyltransferase to stabilize PD-L1 protein. Furthermore, ccRCC exhibits interactive dynamics with tumor-associated macrophages (TAMs). The uridine 5′-diphospho-N-acetylglucosamine (UDP-GlcNAc), which serves as a critical substrate for the O-GlcNAcylation process, facilitates TAMs polarization. In ccRCC cells, HK3 expression is influenced by IL-10 secreted by M2 TAMs. Our study elucidates that HK3-mediated O-GlcNAcylation of EP300 is involved in tumor immune evasion. This finding suggests potential strategies to enhance the efficacy of immune checkpoint blockade therapy.

## Introduction

Renal cell carcinoma (RCC) is a prevalent malignant tumor within the urinary system, with its global incidence increasing by 2% annually [1]. The ccRCC represents the most frequent subtype of renal cancer [2, 3], accounting for approximately 70–80% of renal cancer cases. It is notorious for its lethality when it metastasizes [4, 5]. Despite the application of various advanced diagnostic and surgical approaches, approximately one-third of ccRCC patients experience either recurrence or metastasis [6, 7]. Targeting therapies, such as tyrosine kinase inhibitors (TKIs) and mTOR inhibitors, have demonstrated substantial efficacy as first-line treatments for metastatic RCC [8–10]. Nonetheless, the diagnosis of ccRCC remains challenging, and patient survival rates remain relatively low [11–13]. The quest for new biomarkers and a deeper understanding of their underlying molecular mechanisms is imperative to develop more effective treatment options for ccRCC.

Numerous cancer cells demonstrate increased glycolysis by converting glucose to lactate via pyruvate even when sufficient oxygen is available—a phenomenon known as the Warburg effect [14, 15]. This metabolic reprogramming is crucial in tumorigenesis across various cancer types, making it a viable target for both cancer diagnosis and targeted therapy [16].

O-GlcNAcylation is a post-translational modification (PTM) that entails the attachment of a monosaccharide to the hydroxyl groups of serine or threonine on various nuclear and cytoplasmic proteins [17]. Approximately 3–5% of intracellular glucose is metabolized via the hexosamine biosynthesis pathway (HBP), which is a branch of glycolysis [18–21]. In this process, UDP-GlcNAc acts as the substrate for O-GlcNAcylation [22], a PTM process catalyzed by the enzyme O-GlcNAc transferase (OGT) and can be dynamically reversed by O-GlcNAcase (OGA) [23]. The site-specific O-GlcNAcylation of proteins plays a pivotal role in regulating several critical cellular processes, including transcription, translation, and metabolic reprogramming. This dynamic and reversible modification influences protein function, stability, and interactions, thereby contributing to the intricate control of cellular physiology [17]. It’s worth noting that O-GlcNAcylation intricately interacts with other PTMs, constituting a crucial mechanism for finely tuning intracellular signaling. Recent research indicates that global levels of O-GlcNAcylation are elevated in several types of cancers, yet the precise molecular mechanisms contributing to their pathology remain poorly understood [24–26].

Resisting T cell cytotoxicity is a crucial aspect of tumor development. Immune checkpoint blockade therapy, utilizing neutralizing monoclonal antibodies to inhibit the interaction between PD-1 and PD-L1 and reinvigorate T cell antitumor activity, has emerged as a promising therapeutic strategy, demonstrating significant efficacy in treating various tumor types [27–29]. Numerous monoclonal antibodies targeting PD-1/PD-L1, including Opdivo and Atezolizumab, have been effectively developed and have shown promise as immunotherapeutic agents for treating a range of solid tumors, such as non-small cell lung cancer, breast cancer, and colorectal cancer [30–32]. However, it is critical to acknowledge that the patient response rates to these therapies remain relatively low across various cancer types, and therapeutic resistance often develops as treatment progresses [33–36]. Consequently, there is a need to understand the mechanisms underlying the tumor-specific upregulation of PD-L1 and to develop strategies for disrupting this process [37].

In this study, we reveal a previously unidentified connection between glycolysis and immune evasion in ccRCC. HK3 maintains EP300 protein stability by regulating O-GlcNAcylation levels in ccRCC, thereby promoting PD-L1 expression. We observed dynamic O-GlcNAcylation of EP300 at Ser900, and this site-specific O-GlcNAcylation enhances the stability of EP300 while EP300 also regulating PD-L1 at the transcriptional and protein levels. Inhibition of HK3 leads to a reduction in PD-L1 expression, which consequently restores T-cell cytotoxicity both in vitro and in immunocompetent mice.

## Material and methods

### Cell culture

We obtained the following cell lines, HK2, Jurkat, 786-O, Caki-1, ACHN, Renca, THP-1, and RAW264.7 from ATCC. The cell lines were consistently maintained in either RPMI 1640 medium (Hyclone) or high-glucose DMEM (Hyclone). The culture media included 10% fetal calf serum (Gibco), supplemented with 100 U/mL penicillin and 100 U/mL streptomycin. Cells were incubated in a humidified environment at 37 °C with 5% CO_2_.

### Human specimens

Small samples (approximately 0.5 cm³) of ccRCC tissues, along with corresponding tumor-adjacent tissues from the same patient, were obtained from surgically excised renal specimens. All patients were enrolled at the Second Affiliated Hospital of Harbin Medical University in Harbin, China. Prior to commencing the study, informed consent was secured from each patient. Pathologists at the Second Affiliated Hospital of Harbin Medical University examined and diagnosed these specimens. The research protocol received approval from the Ethical review of clinical research projects of the Medical Ethics Committee of the Second Affiliated Hospital of Harbin Medical University, Harbin, China. The entire experimental process adhered to institutional guidelines and ethical standards.

### Western blot (WB)

About 30 µg of lysed protein was separated using an 8–15% SDS-PAGE system and then transferred onto a nitrocellulose membrane. Subsequently, the membrane was blocked using a solution of 5% bovine serum albumin and 0.1% Tween 20 in Tris-buffered saline (TBST). After blocking, the membrane was incubated with specific antibodies. The antibodies used in this study were sourced from the following suppliers: anti-HK3 antibody (1:1000, Abcam, ab126217), anti-PD-L1 (1:1000, Abcam, antibody ab205921), anti-Tubulin antibody (1:10000, Proteintech, 10068-1-AP),anti-O-GlcNAc antibody (1:1000, Cell Signaling Technology, #9875), anti-OGA antibody (1:1000, Santa Cruz, sc-376429), anti-OGT antibody (1:1000, Santa Cruz,sc-74546), anti-EP300 antibody (1:1000, Santa Cruz, sc-48343), anti-HCFC1 antibody (1:1000, Santa Cruz, sc-390950), anti-NAA15 antibody (1:1000, Santa Cruz, sc-365931), anti-Flag antibody (1:1000, Abways, AB0028), anti-UB antibody (1:1000, Santa Cruz, sc-8017), anti-Histone H3 antibody (1:1000, Cell Signaling Technology, #9715), anti-TFAP2A antibody (1:1000, Santa Cruz, sc-12726), anti-SP1 antibody (1:1000, Santa Cruz, sc-420), anti-Acetyllysine antibody (1:1000, PTM Bio, PTM-105RM), anti-CD206 antibody (1:1000, Santa Cruz, sc-376108), anti-CD163 antibody (1:1000, Santa Cruz, sc-20066), anti-NOS2 antibody (1:1000, Santa Cruz, sc-7271), anti-CD86 antibody (1:1000, Santa Cruz, sc-28347) and anti-IL10 antibody (1:1000, Santa Cruz, sc-8438). Protein visualization was performed using the Odyssey Infrared Imaging System (Bio-Rad).

### Chromatin immunoprecipitation (ChIP)

Cell lysates were initially pre-cleaned using protein A-agarose conjugated normal IgG (sc-2027, Santa Cruz). Subsequently, cell lysates were treated with 2.0 µg of anti-TFAP2A antibody and incubated overnight at 4 °C. For the negative control, IgG was used. To assess the interaction and binding of these factors, specific primer sets, designed for amplifying the target sequences within the PD-L1 promoter regions, were employed for quantitative polymerase chain reaction (qPCR). The products were then subjected to agarose gel electrophoresis for further analysis.

### Quantitative real-time PCR (qRT-PCR)

TRIzol RNA isolation reagents (Invitrogen) were utilized to extract total RNA from cells and tissues. Complementary DNA (cDNA) was synthesized using the all-in-one First Strand cDNA Synthesis Kit (Sevenbio, Beijing, China), and qRT-PCR was conducted using 2× SYBR Green qPCR MasterMix (Sevenbio, Beijing, China) following the standard protocol. Relative expression levels were calculated using the 2 ^-ΔΔCt^ method. The qRT-PCR primer sequences are provided in Supplementary Table 1.

### Protein stability assay

To determine the stability of endogenous proteins, ccRCC cells were treated with 100 μM cycloheximide (CHX, MCE) and harvested at intervals of 0, 1, 2, 4, 8, and 12 h. To assess the stability of exogenous EP300 proteins (both WT and S900A mutant), 786-O cells were transfected with either Flag-WT or Flag-S900A mutant constructs. Afterward, these cells were also treated with 100 μM CHX and collected at the same intervals: 0, 1, 2, 4, 8, and 12 h. The basal expression levels of Flag-EP300 (WT or S900A) at the 0-hour time point were normalized to comparable levels for accurate comparison.

### Immunofluorescence (IF)

Cell and tissue slides were fixed in 4% paraformaldehyde for 25 min. Subsequently, the samples were incubated with the designated primary antibody overnight. Following this, the slides were incubated with a fluorescence-labeled secondary antibody, and nuclei were stained with DAPI (Beyotime). Imaging was performed using a Leica microscope.

### Immunohistochemical staining (IHC)

Tissues from both patients and mice were collected and fixed in formalin. These tissues were subsequently embedded in paraffin blocks and sectioned into slices, each measuring 4 μm in thickness. The primary antibodies were visualized using horseradish peroxidase diaminobenzidine (HRP-DAB) immunostaining.

### Enzyme-linked immunosorbent assay (ELISA)

Human UDP-GlcNAc ELISA kits were procured from Ruifan Biotechnology (RF6166-48). The assays were performed following the manufacturer’s instructions.

### EP300 O-GlcNAcylation site mapping

We entrusted Biomarker Technologies (Beijing, China) to conduct the analysis of O-GlcNAcylation modifications in proteins within the 786-O cell line. The analysis involved several stages, including protein extraction quality control, enzymatic desalting, peptide enrichment, and mass spectrometry. As a result of this comprehensive process, we identified the O-GlcNAcylation site of EP300 is Ser900.

### Co-culture experiment

To investigate the effect of tumor cells on T-cell inactivation, the tumor cells were co-cultured with activated Jurkat T cells expressing PD-1. Jurkat T cells were initially activated with the ImmunoCult Human CD3/CD28 T Cell Activator (Stemcell Technologies). The co-cultures, maintained at a ratio of 5:1 (Jurkat: tumor cell), were incubated for 24 h. To assess the influence of 786-O cells on macrophage polarization following HK3 knockdown, we initiated the process by stimulating THP-1 cells with PMA to generate M0 macrophages. These macrophage cells were then co-cultured with both normal and HK3 knockdown 786-O cells. Co-cultures were maintained at a ratio of 1:4 (THP-1: tumor cell) and were incubated for 48 h.

### LDH release assay

Adherent tumor cells, including 786-O and Caki-1 (4 × 10^4^ cells per well), were seeded in 24-well plates. Subsequently, various treatments were administered. Activated T cells (2 × 10^5^ cells per well) were added to each well at an effector-to-target ratio of 5 and co-cultured with the adherent tumor cells for 24 h. At the conclusion of the incubation, the plates were centrifuged at 400 g for 5 min. Supernatants from each group were collected to perform the LDH release assay (Beyotime). This set of experiments was conducted in triplicate.

### Extracellular acidification rate (ECAR) assay

ECAR was measured using the Glycolysis Stress Test Kit (Agilent Technologies). In summary, 1.0 × 10^4^ cells were seeded in an XF96 plate and allowed to incubate overnight. The culture media were then replaced with XF media and incubated for an additional hour. Glucose (10 mM), oligomycin (1 μM), and 2-deoxyglucose (2-DG) (100 mM) were added to assess the ECAR [21].

### Animals

All animal studies were carried out in accordance with the guidelines set forth by the Animal Care and Use Committee of Harbin Medical University. The mice were randomly divided into different groups (*n* = 4 per group). Approximately 5 × 10^6^ ccRCC cells and shHK3 cells were injected into the flanks of 6-week-old male BALB/c mice. Tumor volumes were measured once a week starting from day 14. Tumor volume was calculated using the formula: “volume = (width^2^) × length/2.” After 36 days post-injection, the mice were euthanized, and tumors were collected and weighed.

Once the tumor volume reached similar sizes, mice bearing tumors of the same type were randomly assigned to different groups and treated with either anti-PD-1 (BioXcell, Clone RMP1–14) (200 μg, i.p., administered on days 0, 3, and 6) or anti-PD-1 in combination with Coroslic acid. Subsequently, mice were euthanized, and tumors were harvested from each animal. The mass of the grafts was determined based on standard procedures.

### Statistical analysis

All data presented in this article are expressed as the mean ± standard deviation (SD) from three independent experiments. Statistical significance was determined using the Student’s t test and two-way ANOVA test. The comparison of linear correlations was determined using Pearson’s correlation coefficient (R). *P*-value less than 0.05 was considered statistically significant. Significance levels are denoted as follows: *p < 0.05, **p < 0.01, ***p < 0.001, ns no significance.

## Results

### Identification of the glycolytic gene HK3 associated with PD-L1

**Fig. 1:**
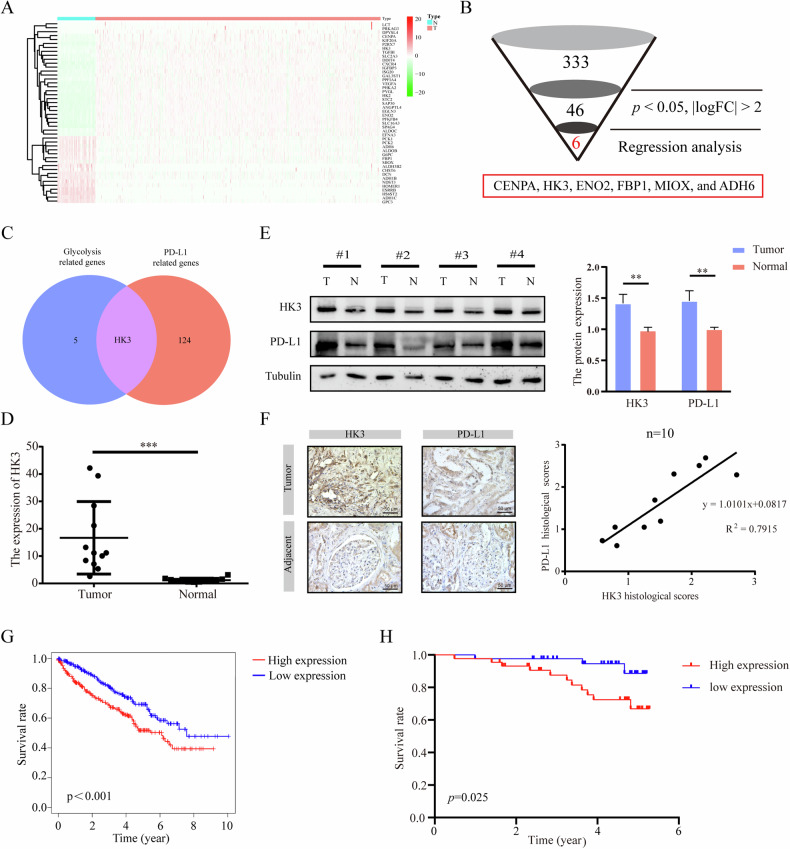
Identification of the glycolytic gene HK3 associated with PD-L1. **A** The heatmap showed the expression distributions of differential GRGs between tumor samples and normal samples of the KIRC. **B** Process of candidate gene selection. **C** Venn diagram of the GRGs and the PD-L1 related genes in KIRC. **D** Quantitative analysis of HK3 expression showing significant increase in tumor tissues compared to the adjacent normal tissues by qRT-PCR (*n* = 12). **E** WB analysis of HK3 protein expression in 4 represented ccRCC tissues and adjacent normal tissues from patients. Quantitation of relative expression levels was shown. **F** IHC stainings with HK3 and PD-L1 were performed in 10 pairs of ccRCC tissues and adjacent normal tissues. Representative images are shown. Scale bar, 50 μm. Correlation analysis of HK3 and PD-L1 expressions, Pearson’s r test. **G** KM survival analysis revealed that an elevated HK3 expression was significantly correlated with a shorter OS in 539 ccRCC patients from the TCGA cohort. **H** KM survival analysis revealed that an elevated HK3 expression was significantly correlated with a shorter OS in 86 ccRCC patients our postoperative follow-up. *p < 0.05, **p < 0.01, ***p < 0.001.

Initially, we obtained transcriptome profiling data from the TCGA database’s KIRC projects, which included 72 normal samples and 539 tumor samples. This data was meticulously annotated using the Ensemble gene transfer format (GTF) file, allowing us to extract an expression matrix of 333 GRGs from TCGA (Supplementary Table 2). We then identified 46 differential GRGs that exhibited significant differential expression in KIRC, distinguishing them from normal samples based on their expression levels (*p* < 0.05, |logFC| > 2), as indicated in Fig. 1A. A univariate Cox regression analysis was performed to assess the prognostic significance of 12 GRGs, illustrated in Supplementary Fig. 1A. From these, 7 GRGs were further selected using LASSO Cox analysis, as depicted in Supplementary Fig. 1B, C. To ascertain the GRGs with the most substantial prognostic value, we employed multiple Cox regression analysis in Supplementary Fig. 1D, E. This process yielded a final selection of 6 GRGs, including CENPA, HK3, ENO2, FBP1, MIOX and ADH6, as shown in Fig. 1B.

To identify genes associated with PD-L1 that play a role in immune evasion, we harnessed the power of UALCAN (https://ualcan.path.uab.edu/). Our search yielded 125 PD-L1 related genes (Supplementary Table 3) (Pearson ≥ 0.3). Cross-referencing these datasets revealed HK3 as the unique glycolytic gene linked to PD-L1, depicted in Fig. 1C.

Data from a comprehensive analysis of the TCGA and CPTAC databases revealed that in ccRCC patients, HK3 expression in primary tumor tissues was markedly elevated compared to normal tissues, at both mRNA and protein levels (Supplementary Fig. 2A). We conducted qRT-PCR, WB and IHC analyses on clinical samples of ccRCC tissues and adjacent normal tissues collected by our team. The results corroborated the database predictions, demonstrating that the mRNA and protein expression levels of HK3 were notably elevated in tumor tissues compared to adjacent normal tissues (Fig. 1D–F). After establishing the differential expression of HK3, Kaplan-Meier (KM) survival analysis revealed significant correlation between elevated HK3 expression and shorter overall survival (OS) in a cohort of 539 ccRCC patients from TCGA (*p* < 0.001) (Fig. 1G). Through our follow-up of 86 ccRCC patients’ postoperative survival, we observed that patients with high HK3 expression had poorer survival outcomes (Fig. 1H). Based on database predictions and validation through clinical samples, we discovered a positive correlation between the expression levels of HK3 and PD-L1. Furthermore, ccRCC patients exhibiting high HK3 expression levels were linked to poorer survival outcomes.

### HK3 promotes tumor immune evasion by upregulating PD-L1 expression

**Fig. 2:**
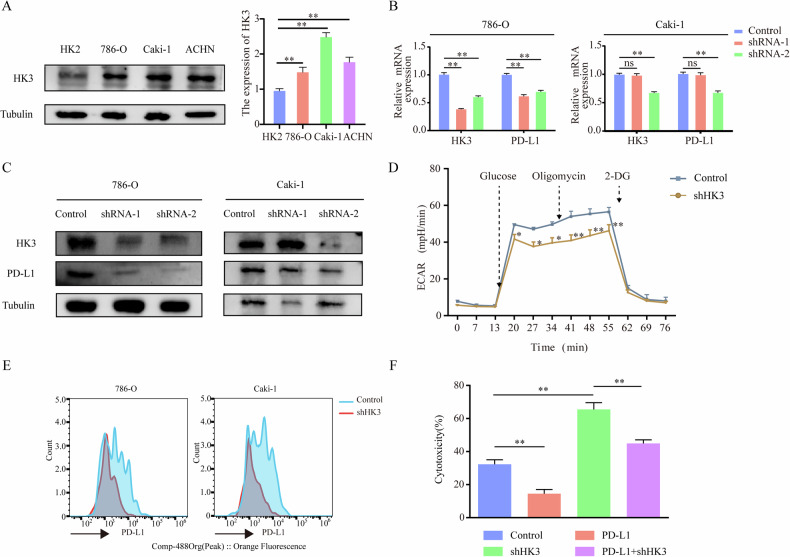
HK3 promotes tumor immune evasion by upregulating PD-L1 expression. **A** WB analysis of HK3 expression using one normal renal cell lines HK2 and three renal cancer cell lines 786-O, Caki-1 and ACHN cells. Quantitation of relative expression levels was shown. The qRT-PCR (**B**), WB (**C**) analysis of HK3 and PD-L1 expression in ccRCC cell lines infected with shControl or shHK3. Tubulin served as an internal reference. The diferences were compared between shControl group and shHK3 group. **D** The ECAR in 786-O cells infected with shControl or shHK3. Each data point was the average of at least three independent measurements. Error bars denote the means ± standard deviations (SD). **E** Flow cytometry analysis showed that membrane surface expression of PD-L1 was reduced in cells transfected with shHK3. **F** The 786-O and Caki-1 cells transfected with indicated shControl and shHK3, were incubated with activated T cells for 16 h and the cytotoxicity was measured by LDH release assay (*n* = 3 independent experiments). *p < 0.05, **p < 0.01, ns no significance.

To elucidate the potential relationship between HK3 and PD-L1, we selected the cell lines as our primary subjects of research. Initially, we analyzed the expression levels of HK3 in renal cell lines, including one normal renal cell line (HK2) and three renal cancer cell lines (786-O, Caki-1, and ACHN). Compared to the normal cell line, all three renal cancer cell lines exhibited higher levels of HK3 expression, mirroring the elevated HK3 expression observed in clinical tumor tissues (Fig. 2A). Subsequently, we selected the 786-O and Caki-1 cell lines for further study. To evaluate the impact of HK3, we knocked down HK3 in these cells using specific shRNA. IF analysis confirmed the successful delivery of shHK3 into the cells (Supplementary Fig. 2B). Furthermore, post-transfection with shHK3, there was a decrease in PD-L1 expression (Fig. 2B, C). As HK3 is a glycolytic gene, we investigated the impact of HK3 depletion on glycolysis levels. In line with our expectations, the depletion of HK3 in 786-O cells led to a significant reduction in the ECAR, indicating a decrease in glycolytic activity (Fig. 2D). It is noteworthy that PD-L1 functions optimally in immune evasion when it is located on the cell membrane interacting with PD-1. To evaluate this, we performed flow cytometry analysis and found that the surface membrane expression of PD-L1 decreased with the transfection with shHK3 (Fig. 2E).

To further explore, we examined if the reduced PD-L1 expression in 786-O and Caki-1 cells, resulting from HK3 depletion, correlated with a diminished immunosuppressive ability against T cells. For this, we used a co-culture system of Jurkat cells with 786-O or Caki-1 cells. We prepared activated T cells by incorporating CD3+/CD28+ (Supplementary Fig. 2C, D). An LDH-based T cell cytotoxicity assay (Fig. 2F) revealed heightened cytolysis in 786-O and Caki-1 cells transfected with shHK3. These findings indicated that HK3 shields ccRCC cells from effector T cell cytotoxicity primarily via PD-L1. This function of HK3 is imperative for ccRCC cells in evading effector T cell cytotoxicity.

### HK3 regulates O-GlcNAcylation levels in ccRCC

**Fig. 3:**
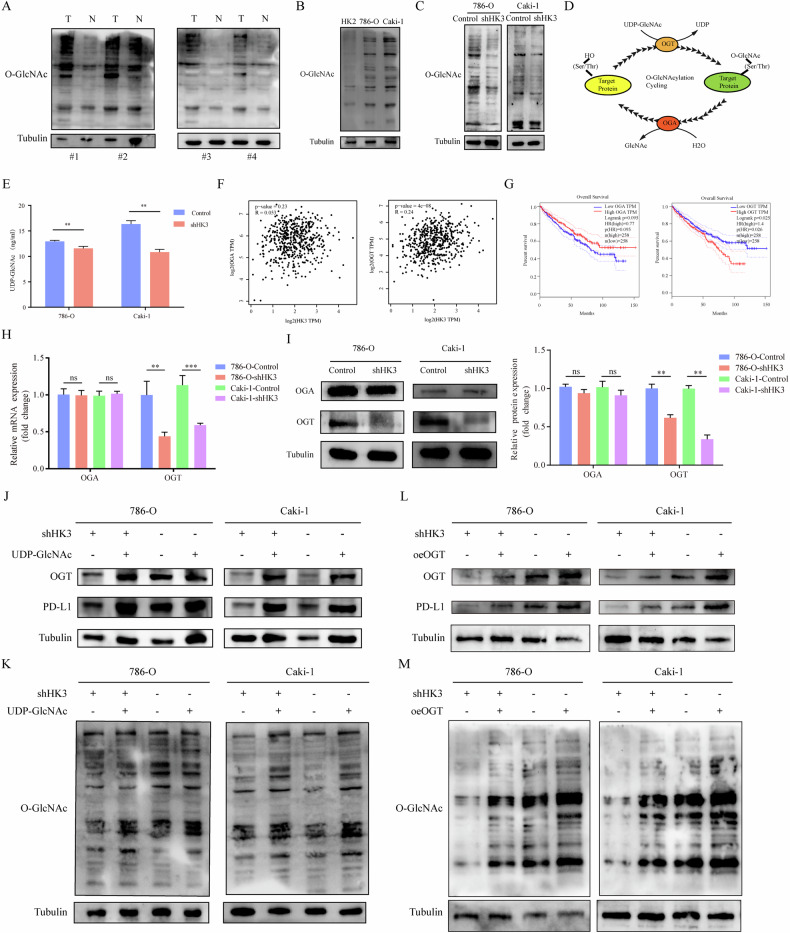
HK3 regulates O-GlcNAcylation levels in ccRCC. **A** The O-GlcNAcylation level of ccRCC tissues and adjacent normal tissues. **B** WB analysis of O-GlcNAcylation expression using one normal renal cell lines HK2 and two ccRCC cell lines 786-O and Caki-1. **C** WB analysis of O-GlcNAcylation in ccRCC cell lines infected with shControl or shHK3. **D** The HBP pathways in cells. **E** The intracellular level of UDP-GlcNAc was detected by ELISA. **F** The correlation between OGA and OGT with HK3. **G** The survival curve shows that there is a significant difference in the expression of OGT on the prognosis of ccRCC. The qRT-PCR (**H**), WB (**I**) analysis of OGA and OGT expression in ccRCC cell lines infected with shControl or shHK3. The ccRCC cells transfected with shControl or shHK3 and then treated with 0 or 20 ng/ml UDP-GlcNAc for another 48 h, OGT, PD-L1 (**J**) and O-GlcNAcylation (**K**) WB analyses were performed using the indicated antibodies. The ccRCC cells transfected with shControl or shHK3 and then treated with control or oeOGT for another 48 h, OGT, PD-L1 (**L**) and O-GlcNAcylation (**M**) WB analyses were performed using the indicated antibodies. *p < 0.05, **p < 0.01, ***p < 0.001, ns no significance.

To delve into the mechanism by which HK3 regulates PD-L1, we initiated our investigation within the glycolytic pathway. The HBP is a metabolic pathway branching from the primary glycolytic pathway [17]. Our research hypothesis revolves around whether HK3 regulates PD-L1 through the HBP pathway. To begin, we validated the O-GlcNAcylation levels in ccRCC tissues in comparison to adjacent normal tissues and observed a notably higher level of O-GlcNAcylation in ccRCC tissues (Fig. 3A). Subsequently, we analyzed O-GlcNAcylation levels using one normal renal cell line (HK2) and two ccRCC cell lines (786-O and Caki-1) (Fig. 3B). All results consistently affirmed significantly higher O-GlcNAcylation levels in ccRCC cells compared to normal renal cells, aligning with the expression pattern of HK3 in ccRCC cells. In a separate experiment, we detected O-GlcNAcylation levels in ccRCC cell lines transfected with shHK3 and found that decreasing HK3 levels caused a decrease in O-GlcNAcylation levels (Fig. 3C).

HBP encompasses three crucial components, namely OGA and OGT as key enzymes and UDP-GlcNAc as a key substrate (Fig. 3D). In comparison to ccRCC cells transfected with shControl, intracellular UDP-GlcNAc levels in shHK3 cells experienced a significant reduction (Fig. 3E). Moreover, we discovered an association between HK3 and OGT (Fig. 3F), and the expression of OGT was correlated with the prognosis of ccRCC patients (Fig. 3G), according to GEPIA (http://gepia.cancer-pku.cn/). This observation aligns with the prognostic significance of HK3 in ccRCC patients (Fig. 1G). Subsequent experiments at both transcriptional and protein levels unveiled a significant reduction in OGT in cells transfected with shHK3, while OGA remained unaffected (Fig. 3H, I). These results indicated that a decrease in HK3 resulted in a substantial reduction in OGT and UDP-GlcNAc, both of which promote O-GlcNAcylation, while OGA, responsible for reducing O-GlcNAcylation, remained unaltered. This substantiates the correlation between O-GlcNAcylation levels and HK3 expression.

We then proceeded to explore whether HK3 influences O-GlcNAcylation levels. Notably, shHK3 suppressed both basal levels and UDP-GlcNAc-elevated PD-L1 and O-GlcNAcylation (Fig. 3J, K), demonstrating that the elevated basal level of tumor HK3, in addition to UDP-GlcNAc-induced HK3, both contributed to PD-L1 expression upon UDP-GlcNAc stimulation. Furthermore, shHK3 suppressed both basal levels and OGT-elevated PD-L1 and O-GlcNAcylation (Fig. 3L, M), signifying that the high basal level of tumor HK3, along with OGT-induced HK3, both played a role in PD-L1 expression following OGT stimulation. These findings unequivocally affirm that HK3 influences the level of O-GlcNAcylation and consequently impacts the expression of PD-L1 by regulating key enzymes (OGT) and substrates (UDP-GlcNAc) within the HBP pathway.

### HK3 affects the O-GlcNAcylation of EP300

**Fig. 4:**
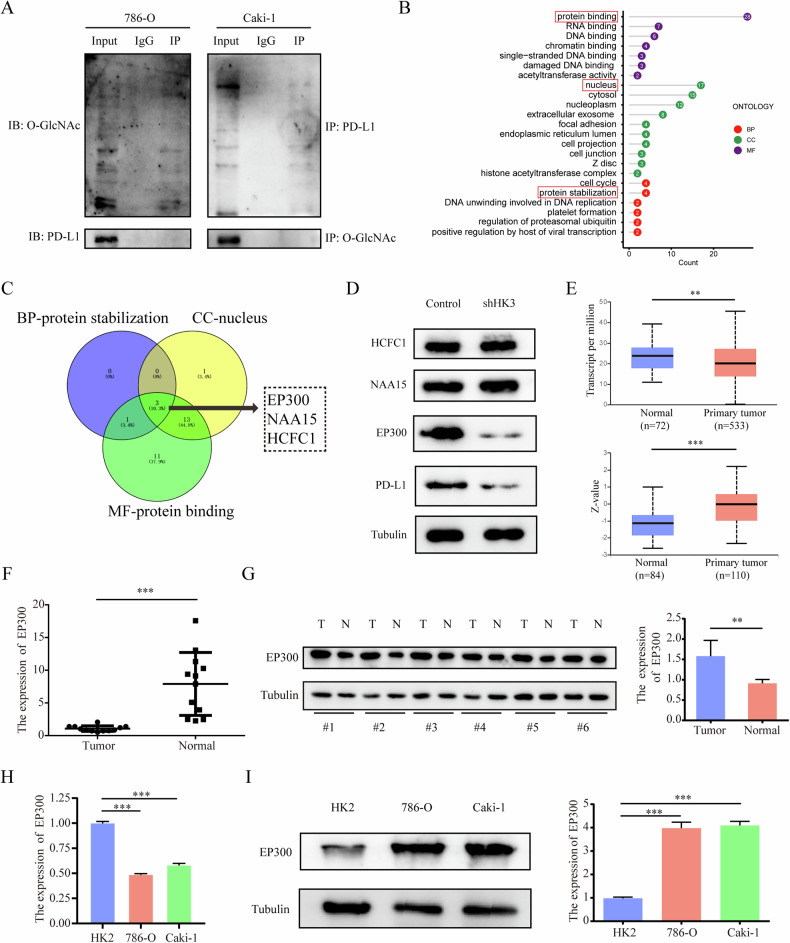
HK3 affects the O-GlcNAcylation of EP300. **A** Co-IP experiment analysis of the interaction between PD-L1 and O-GlcNAcylation in 786-O and Caki-1 cells. **B** The selected 29 genes were used for Gene Ontology-enrichment analysis, BP, CC and MF. **C** Venn diagram demonstrating the overlapping of three enriched gene sets, protein stabilization, nucleus, and protein binding. **D** WB analysis of HCFC1, NAA15, EP300 and PD-L1 expression in ccRCC cell lines infected with shControl and shHK3. **E** The expression of EP300 in ccRCC primary tumor tissues and adjacent normal tissues at the mRNA level (left) from the TCGA database and at the protein level (right) from the CPTAC database. **F** Quantitative analysis of HK3 expression showing significant increase in tumor tissues compared to the adjacent normal tissues by qRT-PCR (*n* = 12). **G** WB analysis of HK3 protein expression in 6 represented ccRCC tissues and adjacent normal tissues from ccRCC patients. Quantitation of relative expression levels was shown. **H** Quantitative analysis of EP300 expression showing significant increase in normal renal cell lines HK2 compared to the two ccRCC cell lines 786-O and Caki-1 by qRT-PCR. **I** WB analysis of EP300 expression using one normal renal cell lines HK2 and two ccRCC cell lines 786-O and Caki-1. Quantitation of relative expression levels was shown. *p < 0.05, **p < 0.01, ***p < 0.001.

As a significant PTM, our initial investigation aimed to determine if O-GlcNAcylation directly alters PD-L1 in ccRCC cells, thereby influencing PD-L1 expression. Our findings, however, indicated that PD-L1 cannot be directly modified by O-GlcNAcylation in 786-O and Caki-1 cell lines (Fig. 4A). Therefore, there must be proteins modified by O-GlcNAcylation to regulate the expression of PD-L1. We detected all proteins modified by O-GlcNAcylation in 786-O, and found a total of 29 proteins with 35 O-GlcNAcylation modification sites (Supplementary Table 4). Gene ontology (GO) analysis of 29 genes were performed using the DAVID (Fig. 4B). Among them, we selected the intersection of the three enriched gene sets with the highest count and lowest p-value in biological processes (BP), cellular components (CC), and molecular functions (MF), namely protein stabilization, nucleus, and protein binding. The intersection genes are EP300, NAA15, and HCFC1 (Fig. 4C). Further verification revealed that only EP300 undergoes changes in expression after HK3 knockdown (Fig. 4D). Therefore, we consider that HK3 may affect the expression of EP300 through O-GlcNAcylation.

EP300 as a pivotal gene known to affect PD-L1 expression in some tumors, excluding ccRCC [38–41]. We observed that the expression of EP300 at the mRNA level was significantly lower in primary tumor tissues compared to adjacent normal tissues in ccRCC patients based on TCGA data. However, the protein level of EP300 presented contrasting results based on CPTAC databases (Fig. 4E). In our own cohort, we noted lower EP300 expression compared to adjacent normal tissues through qRT-PCR analysis, but higher expression at the protein level in tumor tissues, as confirmed by WB analysis (Fig. 4F, G). Analysis of EP300 expression in one normal renal cell line (HK2) and two ccRCC cell lines (786-O and Caki-1) through qRT-PCR (Fig. 4H) and WB (Fig. 4I) revealed consistency with the tissue results, indirectly implying the presence of PTM in EP300.

### O-GlcNAcylation modification at Ser900 stabilizes EP300

**Fig. 5:**
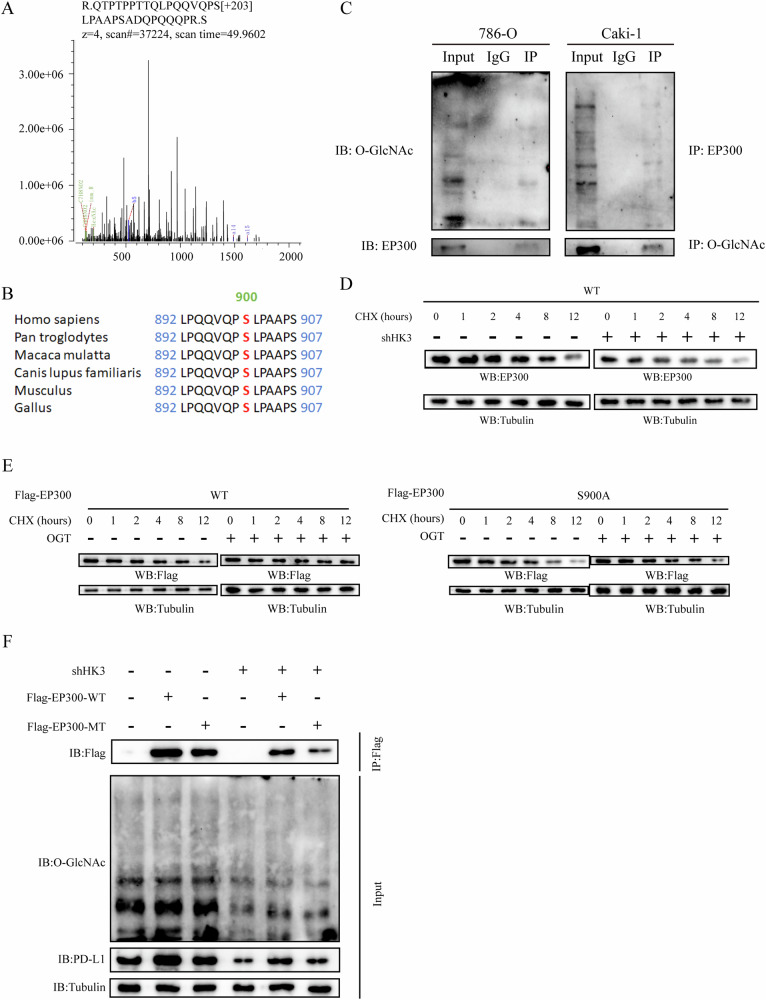
O-GlcNAcylation modification at Ser900 stabilizes EP300. **A** LC-MS analysis of EP300 identified residue Ser900 as the EP300 O-GlcNAcylation site. **B** Cross-species sequence alignment of EP300. **C** Co-IP experiment analysis of the interaction between EP300 and O-GlcNAcylation in 786-O and Caki-1 cells. **D** Analysis of the apparent half-life time of EP300 in 786-O cells transfected with or without shHK3. **E** Analysis of the apparent half-life time of wild type and mutant EP300 in CHX treated cells transfected with indicated vectors. **F** 786-O cells were transfected with shControl or shHK3 were infected with FLAG-EP300-WT and S900A plasmids.

To pinpoint the O-GlcNAcylation site(s) on EP300, we immunoprecipitated Flag-tagged EP300 from 786-O cells and subjected it to mass spectrometry (MS). The results revealed that Ser900 was the O-GlcNAcylation site on EP300 (Fig. 5A). Remarkably, the Ser900 site of EP300 shows a high degree of conservation across vertebrates (Fig. 5B). To confirm the O-GlcNAcylation of EP300, we performed co-immunoprecipitation (Co-IP) verification, as shown in Fig. 5C. O-GlcNAcylation plays a crucial role in promoting protein stability. In our analysis, we observed that transfected with shHK3 hastened the degradation of the EP300 protein, as demonstrated in the CHX chase analysis (Fig. 5D).

To further validate this modification site, we introduced a single mutant Ser900 (S900A) through mutagenesis and transfected it into 786-O cells. Notably, the S900A mutant significantly reduced the half-life of EP300 in comparison to EP300-WT. Additionally, transfection with oeOGT extended the lifespan of EP300-WT but had minimal effects on S900A (Fig. 5E). Although EP300-WT displayed elevated O-GlcNAcylation, O-GlcNAcylation in the S900A mutant was substantially diminished (Fig. 5F). This experimental evidence identifies Ser900 as the site of O-GlcNAcylation on EP300.

### O-GlcNAcylation stabilizes EP300 through suppression of its ubiquitination

**Fig. 6:**
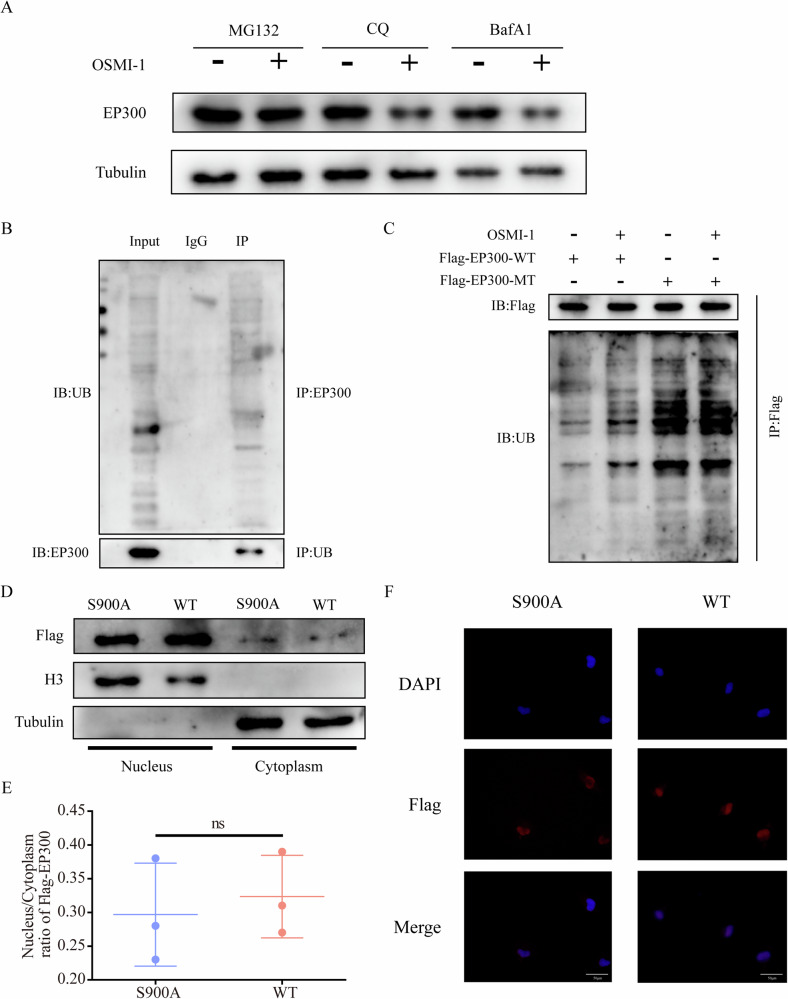
O-GlcNAcylation stabilizes EP300 through suppression of its ubiquitination. **A** 786-O cells were treated with 10 μM MG132, 50 μM chloroquine (CQ) or 100 nM bafilomycin A1 (BafA1) for 6 h before treatment with 20 μM OSMI-1. **B** Co-IP experiment analysis of the interaction between PD-L1 and UB in 786-O cells. **C** 786-O cells were treated with 0 or 20 μM OSMI-1 were infected with FLAG-EP300-WT and S900A plasmids. **D** WB analyses were performed on nuclear and cytoplasm fractions with anti-Flag. **E** The nucleus/cytoplasm ratio of EP300 was quantified. **F** Representative images of subcellular localization of EP300 in 786-O cells. Scale bar, 50 μm. ns no significance.

Eukaryotic cells utilize two primary protein degradation pathways: the ubiquitin-proteasome pathway and the lysosomal pathway. We observed that the sole use of the proteasome inhibitor MG132 significantly counteracted the downregulation of EP300 caused by OSMI-1 treatment (Fig. 6A). Initially, we confirmed that EP300 undergoes ubiquitination, as shown in Fig. 6B. While the EP300-WT reversed ubiquitination, there was a notable reduction in ubiquitination in the EP300-S900A mutant (Fig. 6C). Thus, we suggest that O-GlcNAcylation might influence the stability of EP300 expression by competing with the ubiquitin-proteasome pathway.

Furthermore, we evaluated the possible impact of EP300 O-GlcNAcylation on its subcellular localization. The nuclear/cytoplasmic fractionation assay and IF assay revealed that the mutation altered EP300 expression but had negligible effects on its localization (Fig. 6D–F). These findings suggest that O-GlcNAcylation of EP300 improves its protein stability while minimally affecting its subcellular localization.

### O-GlcNAcylation of EP300 is crucial for PD-L1 expression

We delved into the interaction between EP300 and PD-L1. As indicated in Supplementary Fig. 3A, upon introducing O-GlcNAcylation inhibitors OSMI-1 to 786-O and Caki-1 cells, we observed substantial reductions in both EP300 and PD-L1 protein levels. A T-cell cytotoxicity assay based on LDH release (Supplementary Fig. 3B) revealed heightened cytolysis in 786-O and Caki-1 cells treated with OSMI-1. These outcomes confirm that, as O-GlcNAcylation is inhibited, the levels of EP300 and PD-L1 decrease, further influencing the occurrence of immune evasion following T-cell killing. In a rescue experiment, we further substantiated that HK3 might affect the alterations in PD-L1 through its influence on EP300 (Supplementary Fig. 3C). The above results confirm the existence of O-GlcNAcylation sites in EP300 and its crucial role in the expression of PD-L1.

### EP300 regulates PD-L1 at transcriptional and protein levels

**Fig. 7:**
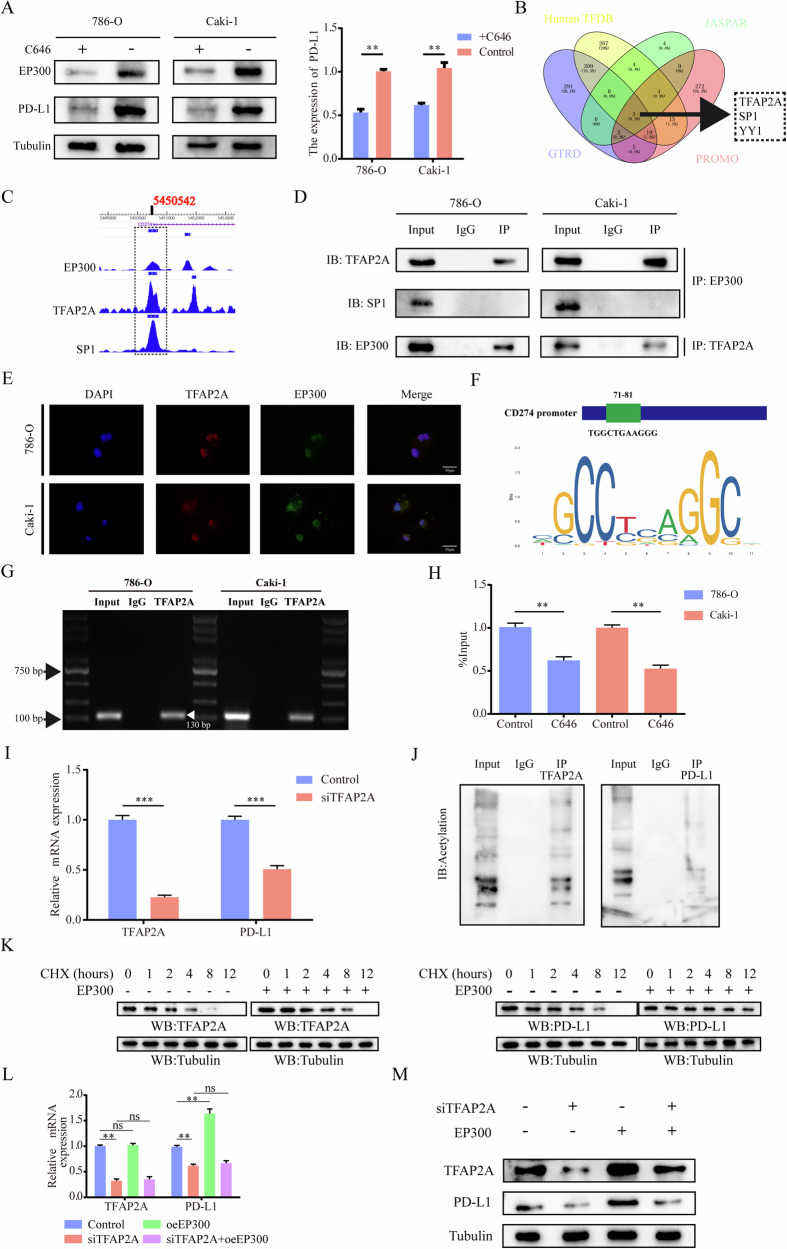
EP300 regulates PD-L1 at transcriptional and protein levels. **A** The WB (left) and qRT-PCR (right) analyses of EP300 and PD-L1 expression in ccRCC cell lines treated with 0 or 20 μM C646. **B** Venn diagram demonstrating the overlapping of four datasets, Human TFDB (http://bioinfo.life.hust.edu.cn/HumanTFDB#!/), JASPAR (https://jaspar.genereg.net/), GTRD (http://gtrd20-06.biouml.org/) and PROMO (https://alggen.lsi.upc.es/cgi-bin/promo_v3/promo/promoinit.cgi?dirDB=TF_8.3) comprised three genes: TFAP2A, SP1, YY1. **C** Depiction of EP300, TFAP2A and SP1 binding peaks at CD274 promoter from Cistrome Data Browser (http://cistrome.org/db/#/). **D** Co-IP experiment analysis of the interaction of EP300 with TFAP2A and SP1 in 786-O and Caki-1 cells. **E** Representative IF images of TFAP2A and EP300 in vitro ccRCC cells. Scale bar, 50 μm. **F** TFAP2A-responsive elements motif sequence identified with JASPAR. **G** ChIP assay confirmed that TFAP2A could directly bind with CD274 (−71 nt to −81 nt). **H** ChIP-qPCR was performed using TFAP2A antibodies after 786-O and Caki-1 were treated with or without C646 (20 μM) (*n* = 3). **I** The qRT-PCR analysis of TFAP2A and PD-L1 expression in ccRCC cell lines infected with siControl or siTFAP2A. **J** Co-IP experiment analysis of the interaction between PD-L1 and KAC in 786-O and Caki-1. **K** Analysis of the apparent half-life time of TFAP2A and PD-L1 in 786-O cells infected with or without oeEP300. The ccRCC cells transfected with siControl or siTFAP2A and then treated with control or oeEP300 for another 48 h, TFAP2A, PD-L1 qRT-PCR (**L**) and WB (**M**) analyses were performed using the indicated antibodies. *p < 0.05, **p < 0.01, ***p < 0.001, ns no significance.

To delve deeper into how EP300 regulates PD-L1, we initially introduced EP300 inhibitors (C646) and observed a positive correlation between PD-L1 and EP300 expression (Fig. 7A). EP300, aside from its role as a histone protein acetyltransferase, can acetylate non-histone proteins and form transcriptional complexes with transcription factors. It acts as a transcriptional co-activator, influencing gene expression through nuclear receptors and other transcription factors [42, 43]. In our investigation, we predicted and analyzed the transcription factors associated with PD-L1 using four different databases. This led to the identification of three transcription factors: TFAP2A, SP1, and YY1 (Fig. 7B). Subsequently, we examined the ChIP-seq data from the Cistrome Data Browser (http://cistrome.org/db/#/) and found overlapping binding sites for TFAP2A, SP1, and EP300 in the promoter region of PD-L1 (Fig. 7C). Further, we conducted Co-IP assays using EP300, TFAP2A, and SP1 and found that only TFAP2A interacted with EP300 in the 786-O and Caki-1 cell lines (Fig. 7D). IF images also confirmed the co-localization of EP300 and TFAP2A in the nucleus (Fig. 7E). These results confirm the co-localization of EP300 and the transcription factor TFAP2A in the nucleus.

To investigate whether TFAP2A directly activates the transcription of PD-L1, we predicted TFAP2A-responsive elements (TREs) in the 2-kb region of the PD-L1 promoter. A rigorously screened TRE (−71 nt to −81 nt) was selected (Fig. 7F). The ChIP assay demonstrated that TFAP2A could effectively bind to the TRE on the PD-L1 promoter region, initiating its transcription (Fig. 7G). Treatment with C646 reduced TFAP2A occupancy at the PD-L1 promoter (Fig. 7H). Additionally, mRNA levels of PD-L1 significantly decreased in the 786-O cell line upon siTFAP2A transfection (Fig. 7I). These results confirm that TFAP2A, as a transcription factor of PD-L1, directly regulates its transcription, while EP300 forms a transcription complex with transcription factors, playing a role in transcriptional co-activation.

Furthermore, in addition to the transcription complex formed by EP300 and TFAP2A, which jointly promotes the transcription of PD-L1, EP300 also acetylates TFAP2A and PD-L1 (Fig. 7J). CHX chase analysis showed that the overexpression of EP300 slowed down the degradation of TFAP2A and PD-L1 proteins (Fig. 7K). After transferring to siTFAP2A and oeEP300, it was observed that EP300 played a protective role in the protein levels of TFAP2A and PD-L1 through changes in transcription and protein levels (Fig. 7L, M). These results confirm that EP300 not only regulates PD-L1 expression at the transcriptional level through TFAP2A but also controls PD-L1 expression at the protein level through acetylation.

### UDP-GlcNAc promotes TAMs to polarize into M2 TAMs

**Fig. 8:**
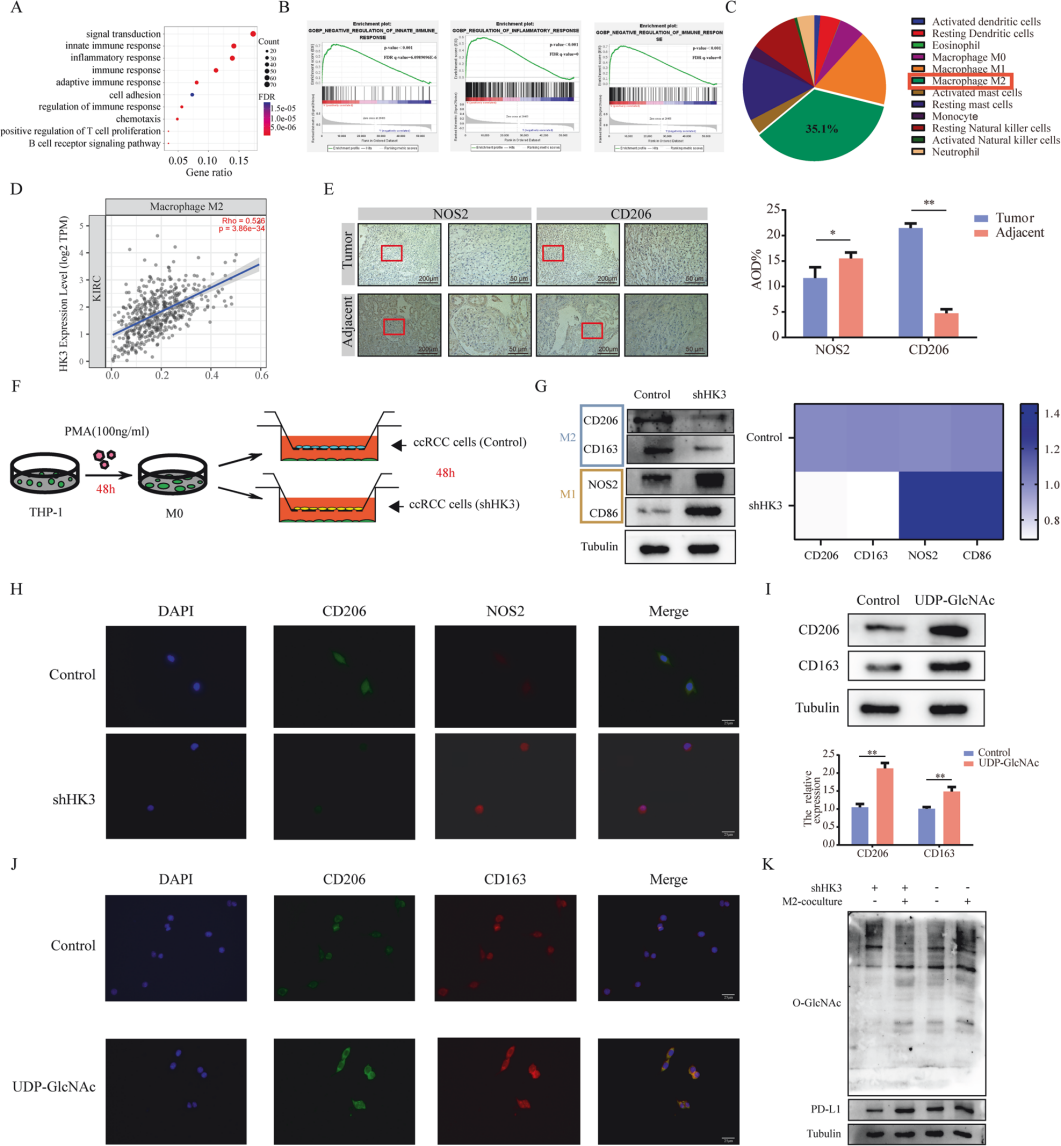
UDP-GlcNAc promotes TAMs to polarize into M2 TAMs. **A** KEGG pathway analysis of HK3 revealed the enriched signaling pathways. **B** The GSEA plot shows a negative correlation between the enrichment of HK3 and immune regulation. **C** Proportion of different types of innate immune cells resided in KIRC tissue. **D** Correlation analysis of HK3 mRNA level and M2 macrophages. **E** Representative IHC staining images showing the expression of NOS2 and CD206 in tumor tissues and adjacent normal tissues (left, scale bar = 200 μm and right, scale bar = 50 μm). **F** The co-culture model of ccRCC cells and macrophages. **G** WB analyses of the M1 macrophage marker (CD86, NOS2) and M2 macrophage marker (CD163, CD206) expression. Quantitation of relative expression levels was shown. **H** Representative IF images of macrophage surface markers CD206 and NOS2 in vitro. Scale bar, 25 μm. **I** WB analyses of the M2 macrophage marker (CD163, CD206) expression. Quantitation of relative expression levels was shown. **J** Representative IF images of macrophage surface markers CD206 and CD163 after co-cultivation with ccRCC cells in vitro. Scale bar, 25 μm. **K** WB analyses of O-GlcNAcylation and PD-L1 in shControl and shHK3 ccRCC cells with or without M2 macrophages-coculture medium. *p < 0.05, **p < 0.01, ***p < 0.001.

Our previous results have confirmed that HK3 plays a role in regulating PD-L1, thereby contributing to immune evasion in tumor cells. We have further analyzed the pathways involved using KEGG and GSEA, and it appears that HK3 is not only involved in specific immunity but also mediates innate immunity within the tumor microenvironment (Fig. 8A, B). The development and immune evasion of tumors depend to varying degrees on the contribution of immune cells in tumor microenvironment (TME). On the contrary, the composition of immune cells in TME also depends to varying degrees on the state of tumor cells. This also provides opportunities for therapeutic interventions. To obtain comprehensive immunogenomic data, we turned to the TCIA (https://tcia.at/home). Analyzing TCIA data, we compared the extent of innate immunity cell infiltration in KIRC. Among all innate immunity cell types, M2 macrophages were the most abundant in KIRC tissues (Fig. 8C). In KIRC, we also observed a moderate positive correlation between the expression level of HK3 and the abundance of M2 macrophages using the TIMER2.0 (http://timer.cistrome.org) (Fig. 8D). Confirmed that HK3 is involved in the interaction between tumor cells and M2 macrophages.

Our IHC analysis of ccRCC tissues and adjacent normal tissues confirmed the enrichment of M2 macrophages in tumor tissues (Fig. 8E). Subsequently, we established a co-culture model involving ccRCC cells and macrophages (Fig. 8F). When we co-cultured 786-O cells transfected with shHK3 and shControl with M0 macrophages, the results demonstrated reduced expression of M2 macrophage-related markers (Fig. 8G). IF images also indicated decreased expression of M2 macrophage markers following the co-culture of M0 macrophages with 786-O cells transfected with shHK3 and shControl (Fig. 8H). We further explored the possibility of a substance that differentially expressed and secreted outside the cell after transfection with shHK3 or shControl. As shown in Fig. 3E, UDP-GlcNAc exhibited differential expression and secretion after shHK3 transfection. To investigate the impact of exogenously added UDP-GlcNAc on the differentiation of M0 macrophages, we conducted experiments. The results confirmed that the exogenous addition of UDP-GlcNAc led to a greater polarization of M0 macrophages towards M2 macrophages, aligning with our earlier expectations (Fig. 8I). IF images also revealed increased expression of M2 macrophage markers after exogenous UDP-GlcNAc addition (Fig. 8J). These findings suggest that HK3 might influence TAM differentiation through the exocrine secretion of UDP-GlcNAc.

We observed that HK3 influences the polarization of macrophages towards the M2 macrophages. This prompted us to investigate whether M2 macrophages, in turn, affect the expression of HK3 in ccRCC cells. To explore this, we co-cultured 786-O cells transfected with shHK3 with the conditioned medium of M2 macrophages. Remarkably, 786-O cells exposed to the M2 macrophages medium exhibited higher levels of PD-L1 and O-GlcNAcylation expression (Fig. 8K). We analyzed the KIRC scRNA-seq dataset (GSE145281), and the t-distributed stochastic neighbor embedding (t-SNE) plot of GSE145281 showed that IL10 is primarily expressed in M2 macrophages (Supplementary Fig. 4A). Furthermore, our WB results confirmed that, compared to M1 macrophages, M2 macrophages express higher levels of IL-10 (Supplementary Fig. 4B). By adding IL-10 to 786-O cells transfected with shHK3 and shControl, we observed an increase in the expression of HK3 and PD-L1 (Supplementary Fig. 4C). These results confirm that IL-10 secreted by M2 macrophages further regulates the expression of HK3 and PD-L1, thus may form a positive feedback loop between M2 macrophages and ccRCC cells.

### Upregulation of PD-L1 by HK3 is required for immune evasion

**Fig. 9:**
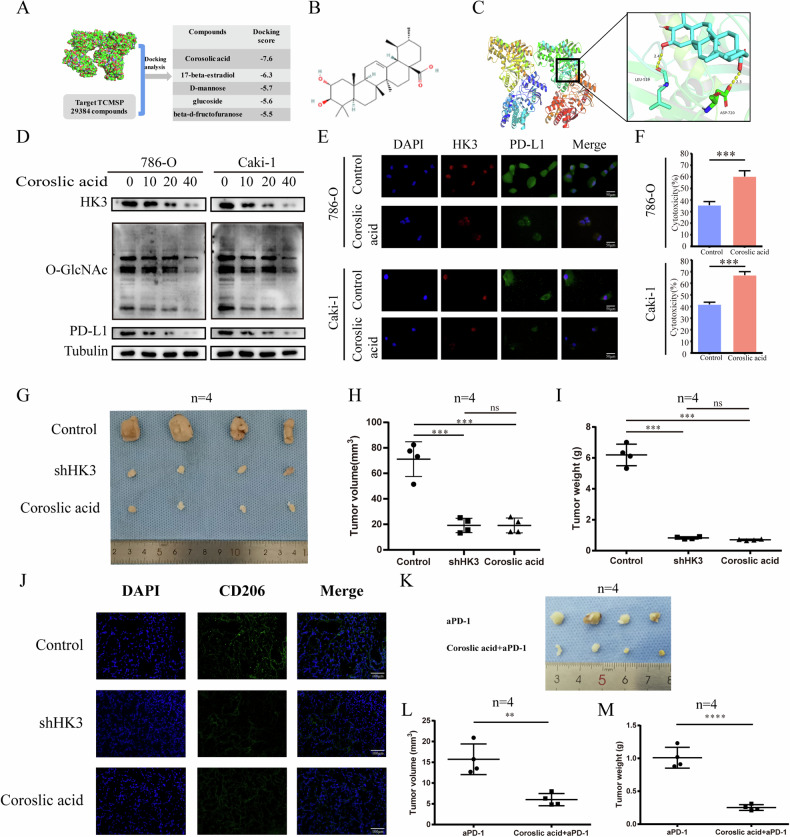
Upregulation of PD-L1 by HK3 is required for immune evasion. **A** Schematic overview of the virtual screening approach based on TCMSP database. **B** Chemical structure of Coroslic acid. **C** Predicted model of Coroslic acid binding to the HK3 as shown by computational docking. **D** Protein expressions of HK3, O-GlcNAcylation and PD-L1 in both Coroslic acid treated 786-O and Caki-1 cell lines under different concentrations. **E** Representative IF images of in vitro ccRCC cells with or without Coroslic acid. Scale bar, 50 μm. **F** The 786-O and Caki-1 cells treated with Coroslic acid, were incubated with activated T cells for 16 h and the cytotoxicity was measured by LDH release assay (*n* = 3 independent experiments). **G** Representative figures of tumor size from mice with various treatments (*n* = 4). The volume (**H**) and weight (**I**) of tumors (*n* = 4). **J** The distribution of M2 macrophages in mice tumor tissues was detected by IF analysis. Scale bar, 100 μm. **K** Representative figures of tumor size from mice with various treatments (*n* = 4). The volume (**L**) and weight (**M**) of tumors (*n* = 4). *p < 0.05, **p < 0.01, ***p < 0.001, ns no significance.

Currently, there is a lack of reported HK3 inhibitors. To address this, we conducted a structure-based virtual screening of approximately 29,000 compounds using the TCMSP database to identify potential HK3 inhibitors. Through a combination pattern analysis, we selected Corosolic acid as the compound with the highest absolute docking score (Fig. 9A–C). Subsequently, we evaluated the effects of Corosolic acid on ccRCC cells. Following treatment with varying concentrations of Corosolic acid (0, 10, 20, and 40 μM) for 48 hours, we observed a dose-dependent decrease in HK3, O-GlcNAcylation, and PD-L1 protein levels in ccRCC cells (Fig. 9D). The IF signal intensities of HK3 and PD-L1 in 786-O and Caki-1 cell lines also exhibited a reduction after Corosolic acid treatment (Fig. 9E). A T cell cytotoxicity assay based on LDH release (Fig. 9F) demonstrated enhanced cytolysis in 786-O and Caki-1 cells treated with Corosolic acid. These results confirm that Corosolic acid, as an inhibitor of HK3, can inhibit HK3 expression in vitro, subsequently affecting the expression of PD-L1 and immune evasion.

Continuing our investigations in vivo, consistent with the in vitro effects, we observed a reduction in tumor volume and weight after HK3 knockdown or treatment with Corosolic acid (Fig. 9G–I). IF analysis revealed a decrease in M2 macrophage markers under both treatments (Fig. 9J). Considering the TME is a complex ecosystem closely associated with responses to checkpoint inhibitor therapy, we investigated the effect of combined therapy. We injected ccRCC cells into the flanks of BALB/C mice and treated them with nivolumab (anti-PD-1) alone or in combination with Corosolic acid. As shown in Fig. 9K–M, the combined therapy resulted in a more significant reduction in tumor volume and weight compared to nivolumab therapy alone. Collectively, these findings support our hypothesis that HK3 can drive tumor cells toward a more M2 macrophage and enhance the antitumor activity of anti-PD-1 treatment through remodeling of the TME.

## Discussion

Although our research slightly overlaps with the existing literature [44], it is not limited to simple validation. We discovered the interaction between HK3 and the HBP pathway and elucidated the specific mechanism of O-GlcNAcylation of EP300 at Ser900, which has not been reported previously. The novelty of our study lies in demonstrating how this specific PTM enhances EP300 stability and subsequently modulates PD-L1 expression, thereby affecting tumor immune evasion in ccRCC. This research aimed to unravel the underlying mechanism by which ccRCC cells induce T cell immune suppression through the HK3/EP300/TFAP2A/PD-L1 axis.

PTMs are essential in regulating the structure and function of numerous metabolic enzymes, making them vital for the regulation of cellular metabolism. Among PTMs, O-GlcNAcylation has been shown to be involved in regulating various biological processes within cells. Notably, this research revealed that HK3 regulates the level of O-GlcNAcylation in ccRCC cells, shedding light on the intricate interplay between glycolysis, PTMs, and immune evasion in ccRCC.

The research findings have unveiled the presence of O-GlcNAcylation modification site, particularly at Ser900, in the EP300 protein. Moreover, it was established that there is an intricate balance between O-GlcNAcylation and ubiquitination, influencing the stability of the EP300 protein. Future investigations may explore whether O-GlcNAcylation of other proteins has a similar impact on the expression of PD-L1. EP300, as a transcription co-activator, engages with multiple transcription regulatory factors and plays a pivotal role in the assembly of basic transcription mechanisms. Furthermore, EP300 is known for its histone acetyltransferase (HAT) activity, which has the potential to modify histone lysine residues as well as non-histone proteins. While the study has highlighted the importance of EP300 in the regulation of PD-L1, the specific mechanisms involving its HAT activity in this context remain a subject for future exploration.

The research also presented UDP-GlcNAc as a key molecule for O-GlcNAcylation and demonstrated its ability to polarize macrophages. However, many questions remain to be answered, including how UDP-GlcNAc is secreted from ccRCC cells into the extracellular space, how macrophages uptake this molecule, and whether it affects the overall O-GlcNAcylation level of macrophages once it enters these immune cells. These aspects represent critical areas for further investigation.

The research highlights the complex interplay between metabolism, tumor development, and immune evasion. Targeting metabolic enzymes for cancer therapy can have significant implications, and the study suggests that HK3, an isoenzyme in the hexokinase family, plays a crucial role in the immune evasion of ccRCC cells by affecting the stability of the EP300 protein and, subsequently, the expression of PD-L1. This discovery provides valuable insights into potential therapeutic targets and combined immunotherapy strategies for ccRCC in the future.

## Conclusions

**Scheme 1:**
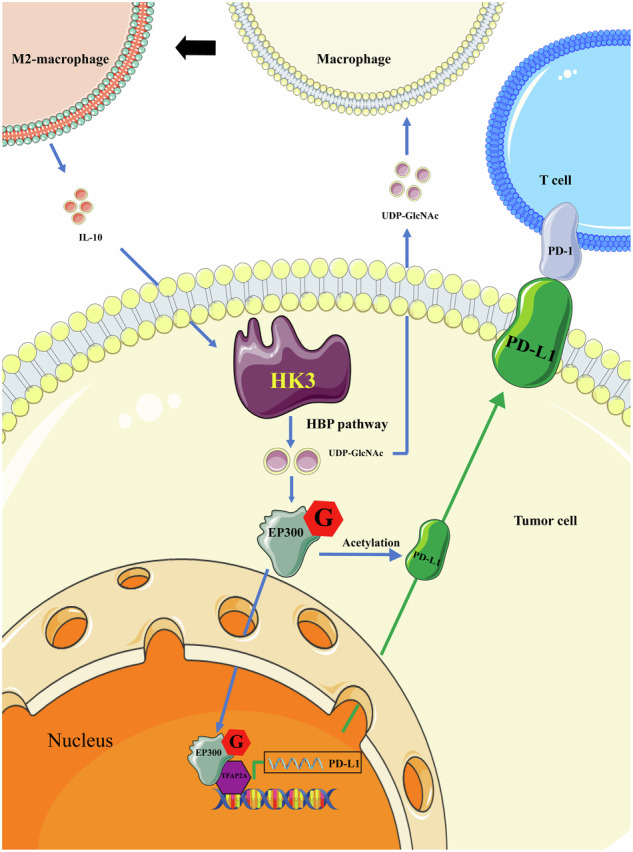
Hexokinase HK3 regulates HBP-related metabolic reprogramming to promote tumor immune evasion and polarize M2 macrophage in ccRCC.

In summary, our findings in Scheme 1 highlight the significance of the glycolytic gene HK3 in affecting O-GlcNAcylation and its impact on the expression of PD-L1 and thus immune evasion. We show that elevated expression of HK3 is associated with an unfavorable prognosis in ccRCC and contributes to PD-L1-mediated immune evasion. HK3 promotes O-GlcNAcylation, stabilizing the EP300 protein, and subsequently enhances the expression of PD-L1 in ccRCC. Furthermore, there is an interaction between TAMs and ccRCC cells. We show that UDP-GlcNAc secreted by ccRCC cells promotes the polarization of TAM to M2 TAM and IL10 secreted by M2 TAM promotes the expression of HK3 in ccRCC cells. Our findings suggest that targeting the HK3/EP300/TFAP2A axis could be a potential therapeutic strategy to enhance the efficacy of PD-L1-mediated immune checkpoint blockade therapy for ccRCC patients.

### Supplementary information

Supplementary information

Original western blot

## Data Availability

Data are available upon reasonable request. All data and material generated in this study are available upon request from the corresponding author.
