# Supplementary material for: Hexokinase HK3-mediated O-GlcNAcylation of EP300: a key regulator of PD-L1 expression and immune evasion in ccRCC
Source: Cell Death Dis. 2024 Aug 23;15(8):613. doi: 10.1038/s41419-024-06921-1 (PMC11343739; doi:10.1038/s41419-024-06921-1)
Supplement: Supplementary file 1 — Supplementary information [file 41419_2024_6921_MOESM1_ESM.pdf]

Supplementary Information

Hexokinase HK3-Mediated O-GlcNAcylation of EP300: A Key Regulator of PD-L1 Expression and Immune Evasion in ccRCC

Supplementary Figures S1-S4

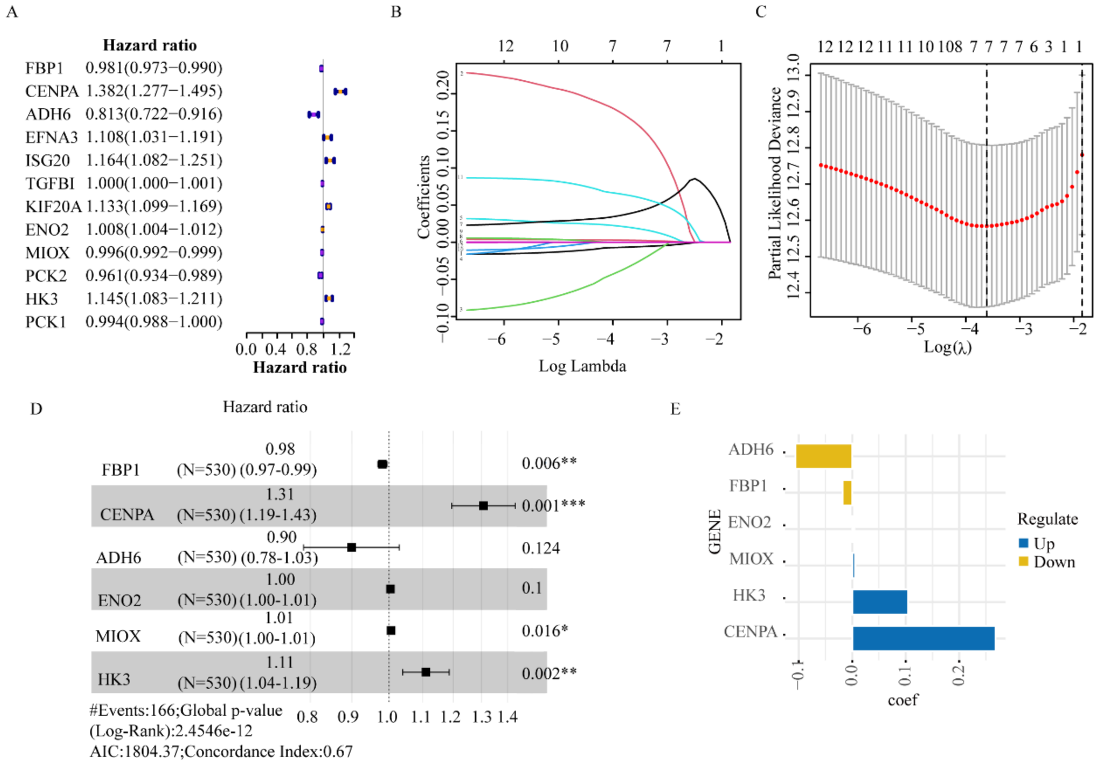

Supplementary Figure1. Process of candidate gene selection.

(A) Univariate COX regression analysis of 12 GRGs. (B) Cross-validation for tuning parameter screening upon LASSO regression analysis. (C) LASSO coefficient profiles for those intersected genes. (D) Forest plot presenting the multiple Cox results. (E) The coefficient of 6 GRGs. *$p < 0.05$, **$p < 0.01$, ***$p < 0.001$.

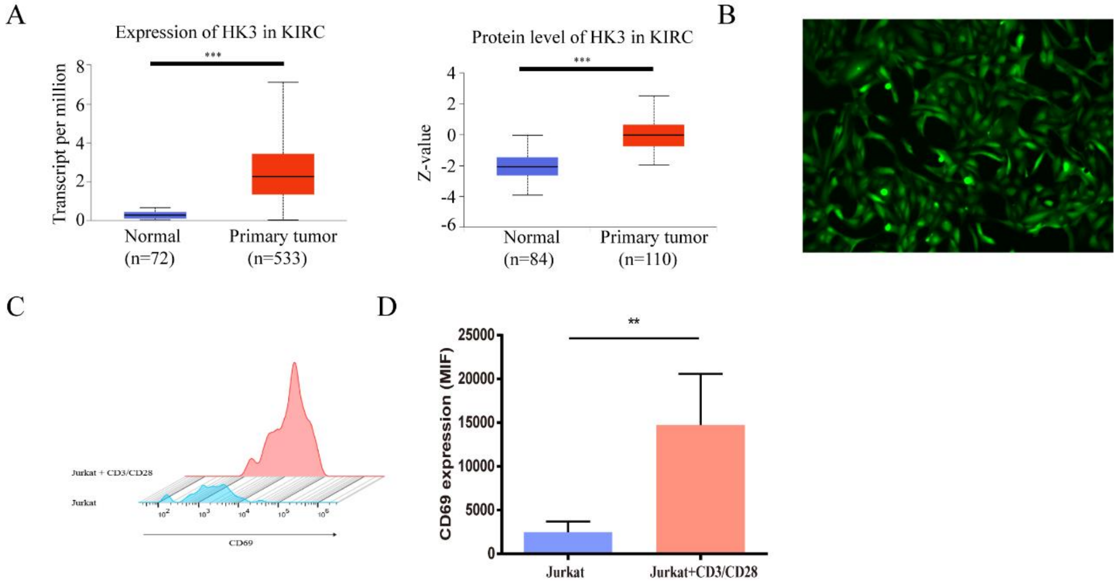

Supplementary Figure2. Differential expression of HK3.

(A) The expression of HK3 in ccRCC primary tumor tissues and adjacent normal tissues at the mRNA level (left) from the TCGA database and at the protein level (right) from the CPTAC database. (B) IF image after transfer to shHK3. (C) The cell surface CD69 expression on T cell is determined using flow cytometry. The Jurkat cells were collected for CD69 detection by flow cytometry. (D) The column charts show the quantitative data of relative CD69 protein expression. *$p < 0.05$, **$p < 0.01$, ***$p < 0.001$.

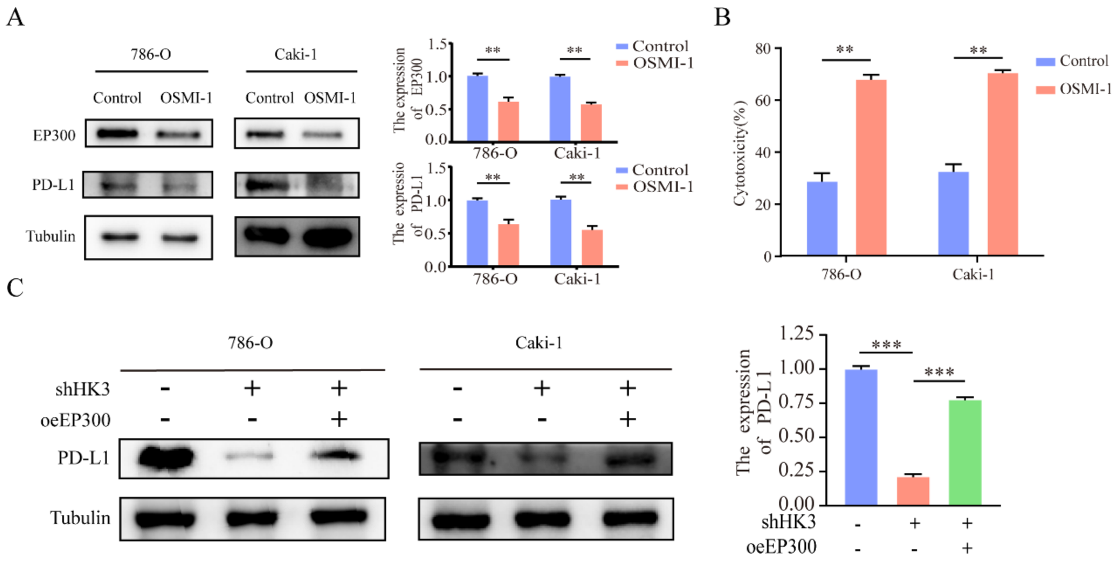

Supplementary Figure3. O-GlcNAcylation of EP300 is crucial for PD-L1 expression.

(A) WB analysis of EP300 and PD-L1 expression in ccRCC cell lines treated with Control and OSMI-1. Quantitation of relative expression levels was shown. (B) The 786-O and Caki-1 cells treated with control and OSMI-1, were incubated with activated T cells for 16 h and the cytotoxicity was measured by LDH release assay (n = 3 independent experiments). (C) WB analysis of expression in ccRCC cell lines infected with shControl, shHK3 and oeEP300. Quantitation of relative expression levels was shown. *$p < 0.05$, **$p < 0.01$, ***$p < 0.001$.

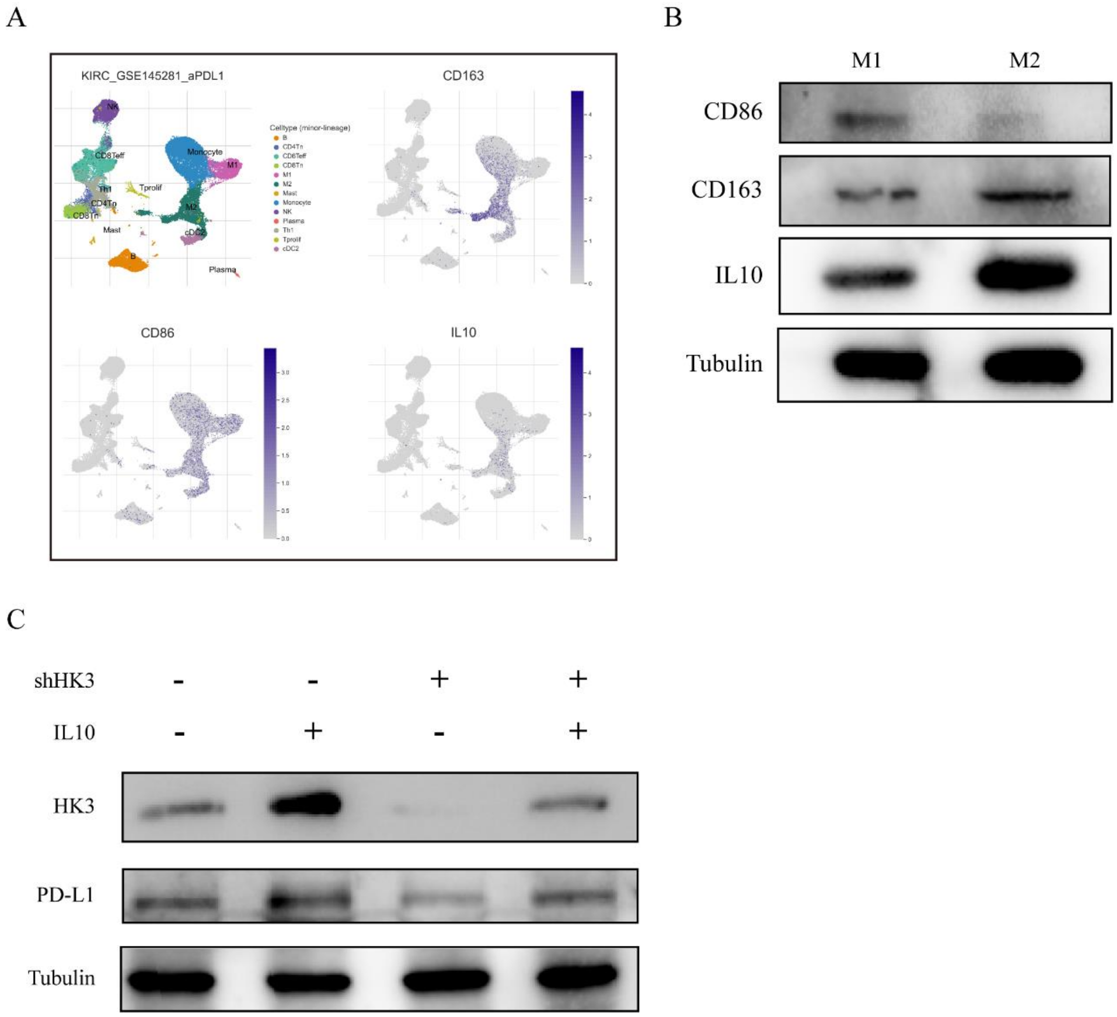

Supplementary Figure4. M2 macrophages secrete IL-10 to regulate the HK3 in ccRCC cells.

(A) t-SNE analysis of 44220 cells of four ccRCC patients (GSE145281). Differential coloring in cell clusters was annotated according to the dominant cell type. Expression of cell-type-specific IL-10, CD163 and CD86 overlaid on the t-SNE space. (B) WB analyses of IL10, the M1 macrophage marker (CD86) and M2 macrophage marker (CD163) expression in M1 macrophages (M1) or M2 macrophages (M2). (C) WB analyses of HK3 and PD-L1 in shControl and shHK3 786-O cells with or without IL-10.

Supplementary Tables S1-S4

Supplementary Table S1. Primer sequences in this study.

Name	Sequence(5'- to -3')	Purpose
HK3 F	5′-AGTTCTTGACCCCAAAGAAA-3′	RT-PCR
HK3 R	5′-TCCAATGACGTGTGTGCGCA-3′	
PD-L1 F	5′-TGCCGACTACAAGCGAATTACTG-3′	RT-PCR
PD-L1 R	5′-CTGCTTGTCCAGATGACTTCGG -3′	
OGA F	5′-AGTGGAGGAAGCTGAGCAAC -3′	RT-PCR
OGA R	5′-TGCAACTTGCCTACTCATCACT -3′	
OGT F	5′-GAGCAGTGCAAGCATACGTC-3′	RT-PCR
OGT R	5′-TGCCAGCCAAATCTCCCCTT-3′	
EP300 F	5′-AAGTATGATCCGTGGCAGTGT-3′	RT-PCR
EP300 R	5′-GTGCTGAAGAGGAGGGGTTT-3′	
TFAP2A F	5′-AGGTCAATCTCCCTACACGAG-3′	RT-PCR
TFAP2A R	5′-GGAGTAAGGATCTTGCGACTGG-3′	
TFAP2A F	5′-AAGGTTTTATCAGTTCGCAG-3′	ChIP
TFAP2A R	5′-CCTCATTAGCATATCAACAAT-3′	

Supplementary Table S2. Glycolysis-related genes.

SLC2A3	PKP2	GNPDA2	PRKACA	GYS2	PLOD1
CACNA1H	LDHAL6B	FBP1	MED24	SEH1L	PPP2CB
PDHA1	PKM	PCK2	ZBTB7A	PGK1	TPST1
HDLBP	TPR	NUP98	PRXL2C	CENPA	PHKA2
CBFA2T3	SEC13	PRKAG1	PPP2CA	CDK1	AKR1A1
GCLC	TFF3	SPAG4	HAX1	CASP6	GLRX
POM121C	ADH1A	GALM	PGM1	ENTPD5	PRKACB
GYS1	COL5A1	P2RX7	GLCE	CHST1	LDHC
SDC2	G6PD	HDAC4	SLC2A1	JMJD8	FBP2
PPARGC1A	GPR87	GMPPA	BIK	GALK1	PCK1
PDK3	TPI1	ENO3	IDUA	NUP107	SOD1
ALDH3A1	CHST6	NDC1	MXI1	OGT	CHST12
PAM	PGP	COG2	PGAM1	CTH	NANP
ALDH3A2	VEGFA	GPC3	ALDOB	AGRN	DHTKD1
CYB5A	BPNT1	ENO1	ZBTB20	ALDH9A1	ADH1B
UGP2	PGM2	XYLT2	FKBP4	CLN6	MPC2
NUP62	AC010618.1	RPE	SLC2A2	EGLN3	ARTN
NUP210	VCAN	SDHC	ACTN3	STMN1	NUP205
TKTL1	HS2ST1	NUP43	PFKFB4	PYGL	EXT2
CLDN9	PGAM2	HS6ST2	GOT2	MPI	PFKM
NDST3	ANGPTL4	GPC1	LCT	POLR3K	NUP214
ABCB6	LDHA	ME1	TREX1	CXCR4	NUP153
COPB2	EXT1	IL13RA1	GMPPB	PFKL	ECD
PGAM4	ISG20	GALK2	STAT3	CLDN3	INSR
CHPF	AC016586.1	PPP2R5D	AAAS	PSMC4	MDH2
RRAGD	AC074143.1	CHST2	AGL	AK4	ALDH2
KIF20A	GFPT1	PKLR	B4GALT2	NUP133	GNE
MIF	PRKAG2	ADPGK	B4GALT1	KDELR3	MYOG
NUP85	MPC1	PPP2R1A	PRPS1	DCN	NUPL2
NT5E	GNPDA1	ENO4	DEPDC1	GCKR	SAP30
ACSS1	CAPN5	AK3	SLC25A13	PRKAG3	PDHB
SRD5A3	TXN	GAPDH	RBCK1	OGDH	HK1
QSOX1	MDH1	GPC4	MLXIPL	ADH6	STC2
GAPDHS	PPFIA4	G6PC2	PFKFB1	ENO2	
PPIA	DLAT	VLDLR	B3GAT3	ARNT	
ADORA2B	PPARA	DLD	HOMER1	IGF1	
NCOR1	PLOD2	PRKAA2	ADH7	HMMR	
GPD1	TGFA	ALG1	TALDO1	PYGB	
ADH4	KIF2A	PGK2	SLC35A3	TIGAR	
CHPF2	RANBP2	PRKAA1	PDHA2	HIF1A	
GOT1	FAM162A	GCK	NUP50	HK3	

MET	STC1	RARS	PFKP	SLC37A4	
NSDHL	CHST4	EGFR	NUP58	NUP155	
GALE	IDH1	SOX9	PMM2	TPBG	
SLC2A5	NUP35	ALDH1A3	HK2	ALDOC	
MERTK	INS	IRS2	BPGM	TGFBI	
ME2	ALDH1B1	MIOX	CD44	NUP93	
PRKACG	DPYSL4	DDIT4	ACSS2	HKDC1	
SLC25A10	G6PC	DSC2	NDUFV3	EFNA3	
AURKA	ALDOA	GUSB	IGFBP3	PAXIP1	
ALDH7A1	NUP37	TSTA3	ELF3	LDHAL6A	
RAE1	ADH5	ANG	NUP54	ANKZF1	
LDHB	POM121	ALDH3B2	SDC3	NASP	
PFKFB3	NUP188	ALDH3B1	FUT8	ESRRB	
P4HA1	ZNF292	B3GNT3	PFKFB2	B4GALT7	
PC	LHX9	CITED2	SLC2A4	ERO1A	
HSPA5	HTR2A	NUP88	SDC1	LHPP	
B3GALT6	PGM2L1	OGDHL	ADH1C	IER3	
B3GAT1	NUP160	B4GALT4	PPP2R1B	GPI	
GAL3ST1	SLC16A3	P4HA2	ARPP19	EIF6	

Supplementary Table S3. PD-L1 related genes.

Genes	Pearson Correlation Coefficient
KANK1	0.9
PGAM2	0.7
IL13RA2	0.68
LTBP1	0.66
CLDN15	0.64
JMJD6	0.62
SEMA3C	0.61
ADAM8	0.6
KIAA0020	0.59
TUBGCP2	0.59
UHRF2	0.54
RTTN	0.5
POP1	0.48
ADAMDEC1	0.47
RCL1	0.47
RIPK2	0.46
LAT	0.45
HNF4G	0.45
SPHK1	0.45
TSPAN13	0.45
FERMT3	0.45
3-Mar	0.44
SEC14L2	0.44
SNHG12	0.44
RPTOR	0.43
FAM54A	0.41
P4HA3	0.41
IARS	0.41
GLDC	0.41
NPEPL1	0.41
P2RY2	0.41
PLOD1	0.41
CXCL13	0.4
RASA3	0.4
GPR68	0.4
UBE2C	0.4
RRM2	0.39
HTRA1	0.39
HTATIP2	0.39
FMNL2	0.39

B3GNT5	0.39
DCBLD1	0.39
MCM4	0.38
ARHGEF5	0.38
MTHFD2	0.38
WNK4	0.38
PDGFC	0.38
LMNB1	0.37
HK3	0.37
OSGIN2	0.37
STIP1	0.37
C20orf20	0.37
ARHGAP11B	0.36
FBXO5	0.36
TNFSF4	0.36
AHR	0.36
CENPE	0.36
OR2A7	0.36
MARCO	0.36
RAD54L	0.36
NVL	0.35
KIF18B	0.35
DFNA5	0.35
UBA6	0.35
CDCP1	0.35
UHRF1	0.35
AURKA	0.35
SPC24	0.35
TMEM164	0.35
ARHGEF35	0.34
SKA3	0.34
CKLF	0.34
PLCB3	0.34
AVL9	0.34
NCAPH	0.34
XPO5	0.34
MOCOS	0.34
DLEU2	0.34
TRIM65	0.34
VLDLR	0.34
PLK4	0.34
HGF	0.34
MTBP	0.34

APBA2	0.33
IGFBP2	0.33
CCNF	0.33
POLD1	0.33
CENPP	0.33
EPR1	0.33
JAK2	0.33
CCNA2	0.33
CDCA5	0.33
EFNA5	0.33
CENPO	0.33
UBE2O	0.33
RACGAP1	0.32
MKI67	0.32
TNIP2	0.32
WDFY2	0.32
GPR174	0.32
NT5E	0.32
MAD2L1	0.32
BRI3BP	0.32
C13orf34	0.32
LRFN4	0.32
CKAP2L	0.32
PLK1	0.32
MTHFD1L	0.31
PKMYT1	0.31
YWHAG	0.31
LOC441294	0.31
TXNDC17	0.31
SRPX2	0.31
CCR5	0.31
TMEM184A	0.31
IQGAP3	0.31
THOC4	0.31
CD80	0.31
SFRS2	0.3
CLSPN	0.3
MAST2	0.3
BUB1	0.3
NTNG2	0.3
RAN	0.3
NSMAF	0.3

Supplementary Table S4. O-GlcNAcylation modification sites.

Protein.Name	Position.in.protein	Sequence	Glycans	Modified. AAs	Mod. Summary	intensity_in_maxquant
>sp|O14974|MYPT1_HUMAN Protein phosphatase 1 regulatory subunit 12A OS=Homo sapiens OX=9606 GN=PPP1R12A PE=1 SV=1	396	K.TKPLASVTNANTSSTQAAPVAVT[+203.079]TPTVSSGQATPTSPIK.K	HexNAc(1)	T	T[+203]	14343000
>sp|O14974|MYPT1_HUMAN Protein phosphatase 1 regulatory subunit 12A OS=Homo sapiens OX=9606 GN=PPP1R12A PE=1 SV=1	645	K.DSVPTAVTIPVAPTVVNAAAST[+203.079]TTLTTTTAGTVSSTTEVR.E	HexNAc(1)	T	T[+203]	0
>sp|O60563|CCNT1_HUMAN Cyclin-T1 OS=Homo sapiens OX=9606 GN=CCNT1 PE=1 SV=1	15	R.WYFT[+203.079]R.E	HexNAc(1)	T	T[+203]	8782200
>sp|P01024|CO3_HUMAN Complement C3 OS=Homo sapiens OX=9606 GN=C3 PE=1 SV=2	706	R.FS[+203.079]C[+57.021]QR.R	HexNAc(1)	C, S	C[+57], S[+203]	9596000
>sp|P12814|ACTN1_HUMAN Alpha-actinin-1 OS=Homo sapiens OX=9606 GN=ACTN1 PE=1 SV=2	754	K.GISQEQMNEFRAS[+203.079]FNHFDR.D	HexNAc(1)	S	S[+203]	25972000

>sp|P13489|RINI_HUMAN Ribonuclease inhibitor OS=Homo sapiens OX=9606 GN=RNH1 PE=1 SV=2	440	R.QPGC[+57.021]LLEQLVLYDIYWS[+203.079]EEMEDR.L	HexNAc(1)	C, S	C[+57], S[+203]	39570000
>sp|P23246|SFPQ_HUMAN Splicing factor, proline- and glutamine-rich OS=Homo sapiens OX=9606 GN=SFPQ PE=1 SV=2	158	K.PVVAQGPGPAPGVGSAPPASSSAPPATPPTSGAPPGSGPGPTPTPPPAVT[+203.079]SAPPGAPPPTPPSSGVPTTPPQAGGPPPPPAAVPGPGPGPK.Q	HexNAc(1)	T	T[+203]	0
>sp|P27694|RFA1_HUMAN Replication protein A 70 kDa DNA-binding subunit OS=Homo sapiens OX=9606 GN=RPA1 PE=1 SV=2	258	K.VYYFS[+203.079]K.G	HexNAc(1)	S	S[+203]	19602000
>sp|P51610|HCFC1_HUMAN Host cell factor 1 OS=Homo sapiens OX=9606 GN=HCFC1 PE=1 SV=2	651	K.SGTVTVAQQAQVVT[+203.079]TVVGGVTK.T	HexNAc(1)	T	T[+203]	17876000
>sp|P54727|RD23B_HUMAN UV excision repair protein RAD23 homolog B OS=Homo sapiens OX=9606 GN=RAD23B PE=1 SV=1	97	K.AVSTPAPATTQQSAPASTT[+203.079]AVTSSTTTTVAQAPTPVPALAPTSTPASITPASATASSEPAPASAAK.Q	HexNAc(1)	T	T[+203]	19064000
>sp|P54727|RD23	125	K.AVSTPAPATTQQSAPASTTAVTSSTTTTVAQAPTPVPALAPTSTPAS[+203.079]IT	HexN	S	S[+20	14655000

B_HUMAN UV excision repair protein RAD23 homolog B OS=Homo sapiens OX=9606 GN=RAD23B PE=1 SV=1		PASATASSEPAPASAAK.Q	Ac(1)		3]	
>sp|Q01082|SPTB2_HUMAN Spectrin beta chain, non-erythrocytic 1 OS=Homo sapiens OX=9606 GN=SPTBN1 PE=1 SV=2	2323	K.HEVSASTQSTPAS[+203.079]SR.A	HexNAc(1)	S	S[+203]	54895000
>sp|Q02809|PLOD1_HUMAN Procollagen-lysine,2-oxoglutarate 5-dioxygenase 1 OS=Homo sapiens OX=9606 GN=PLOD1 PE=1 SV=2	342	K.HMRLFIHNHEQHHKAQVEEFLAQHGS[+203.079]EYQSVK.L	HexNAc(1)	S	S[+203]	14830000
>sp|Q03001|DYST_HUMAN Dystonin OS=Homo sapiens OX=9606 GN=DST PE=1 SV=4	5038	K.EVVTDENKS[+203.079]LIQK.V	HexNAc(1)	S	S[+203]	15262000
>sp|Q09472|EP300_HUMAN Histone acetyltransferase p300 OS=Homo sapiens OX=9606 GN=EP300 PE=1 SV=2	900	R.QTPTPPTTQLPQQVQPS[+203.079]LPAAPSADQPQQQPR.S	HexNAc(1)	S	S[+203]	29836000
>sp|Q14566|MCM6_HUMAN	219	R.GS[+203.079]IPR.S	HexNAc(1)	S	S[+203]	12778000

DNA replication licensing factor MCM6 OS=Homo sapiens OX=9606 GN=MCM6 PE=1 SV=1						
>sp|Q14914|PTGR1_HUMAN Prostaglandin reductase 1 OS=Homo sapiens OX=9606 GN=PTGR1 PE=1 SV=2	45	K.TAELPPLKNGEVLLEALFLT[+203.079]VDPYM[+15.995]R.V	HexNAc(1)	M, T	M[+16], T[+203]	23186000
>sp|Q15293|RCN1_HUMAN Reticulocalbin-1 OS=Homo sapiens OX=9606 GN=RCN1 PE=1 SV=1	55	R.VVRPDSELGERPPEDNQS[+203.079]FQYDHEAFLGK.E	HexNAc(1)	S	S[+203]	10777000
>sp|Q16822|PCKGM_HUMAN Phosphoenolpyruvate carboxykinase [GTP], mitochondrial OS=Homo sapiens OX=9606 GN=PCK2 PE=1 SV=4	309	R.YVAAAFPSAC[+57.021]GKT[+203.079]NLAMM[+15.995]R.P	HexNAc(1)	C, M, T	C[+57], M[+16], T[+203]	17610000
>sp|Q5JRA6|TGO1_HUMAN Transport and Golgi organization protein 1 homolog OS=Homo sapiens OX=9606 GN=MIA3 PE=1 SV=1	1096	R.VIGDTHAS[+203.079]EVSQKPNTEK.D	HexNAc(1)	S	S[+203]	20567000
>sp|Q71RC2|LARP4_HUMAN La-	677	K.DYS[+203.079]GFR.G	HexNAc(1)	S	S[+203]	0

related protein 4 OS=Homo sapiens OX=9606 GN=LARP4 PE=1 SV=3						
>sp|Q8IWE5|PKHM2_HUMAN Pleckstrin homology domain-containing family M member 2 OS=Homo sapiens OX=9606 GN=PLEKHM2 PE=1 SV=2	785	K.EGMLHYKAGTS[+203.079]YLGK.E	HexNAc(1)	S	S[+203]	0
>sp|Q93052|LPP_HUMAN Lipoma-preferred partner OS=Homo sapiens OX=9606 GN=LPP PE=1 SV=1	198	K.STLKPQPAPQAGPIPVAPIGT[+203.079]LKPQPQPVPASYTTASTSSR.P	HexNAc(1)	T	T[+203]	13442000
>sp|Q9BS26|ERP44_HUMAN Endoplasmic reticulum resident protein 44 OS=Homo sapiens OX=9606 GN=ERP44 PE=1 SV=1	369	R.EFHHGPDPTDT[+203.079]APGEQAQDVASSPPESSFQK.L	HexNAc(1)	T	T[+203]	59648000
>sp|Q9BXJ9|NAA15_HUMAN N-alpha-acetyltransferase 15, NatA auxiliary subunit OS=Homo sapiens OX=9606 GN=NAA15 PE=1 SV=1	729	K.DLSDT[+203.079]VR.T	HexNAc(1)	T	T[+203]	7206800
>sp|Q9C0C9|UBE2O_HUMAN	977	K.T[+203.079]FEDR.M	HexNAc(1)	T	T[+203]	0

(E3-independent) E2 ubiquitin-conjugating enzyme OS=Homo sapiens OX=9606 GN=UBE2O PE=1 SV=3						
>sp|Q9NQC3|RTN4_HUMAN Reticulon-4 OS=Homo sapiens OX=9606 GN=RTN4 PE=1 SV=2	150	K.LPEDDEPPARPPPPPPAS[+203.079]VSPQAEPVWTPPAPAPAAPPSTPAAPK.R	HexNAc(1)	S	S[+203]	14378000
>sp|Q9NQC3|RTN4_HUMAN Reticulon-4 OS=Homo sapiens OX=9606 GN=RTN4 PE=1 SV=2	152	K.LPEDDEPPARPPPPPPASVS[+203.079]PQAEPVWTPPAPAPAAPPSTPAAPK.R	HexNAc(1)	S	S[+203]	14378000
>sp|Q9NQC3|RTN4_HUMAN Reticulon-4 OS=Homo sapiens OX=9606 GN=RTN4 PE=1 SV=2	160	K.LPEDDEPPARPPPPPPASVSPQAEPVWT[+203.079]PPAPAPAAPPSTPAAPK.R	HexNAc(1)	T	T[+203]	0
>sp|Q9UG63|ABCF2_HUMAN ATP-binding cassette sub-family F member 2 OS=Homo sapiens OX=9606 GN=ABCF2 PE=1 SV=2	230	K.DFS[+203.079]GGWRMR.V	HexNAc(1)	S	S[+203]	10338000
>sp|Q9UHD9|UBQL2_HUMAN Ubiquilin-2 OS=Homo sapiens OX=9606 GN=UBQLN2	124	K.SQNRPQGQSTQPSNAAGTNTT[+203.079]SASTPR.S	HexNAc(1)	T	T[+203]	77837000

PE=1 SV=2						
>sp|Q9UHR5|S30BP_HUMAN SAP30-binding protein OS=Homo sapiens OX=9606 GN=SAP30BP PE=1 SV=1	239	K.GTTTNATSTT[+203.079]TTTASTAVADAQK.R	HexNAc(1)	T	T[+203]	24002000
>sp|Q9UHR5|S30BP_HUMAN SAP30-binding protein OS=Homo sapiens OX=9606 GN=SAP30BP PE=1 SV=1	237	K.GTTTNATS[+203.079]TTTTTASTAVADAQK.R	HexNAc(1)	S	S[+203]	24002000
>sp|Q9UHR5|S30BP_HUMAN SAP30-binding protein OS=Homo sapiens OX=9606 GN=SAP30BP PE=1 SV=1	244	K.KGTTTNATSTTTTTAS[+203.079]TAVADAQK.R	HexNAc(1)	S	S[+203]	9946700
>sp|Q9UQ80|PA2G4_HUMAN Proliferation-associated protein 2G4 OS=Homo sapiens OX=9606 GN=PA2G4 PE=1 SV=3	206	K.TIIQNPT[+203.079]DQQKK.D	HexNAc(1)	T	T[+203]	15510000
