# Supplementary figures and images for: Hexokinase HK3-mediated O-GlcNAcylation of EP300: a key regulator of PD-L1 expression and immune evasion in ccRCC
Source: Cell Death Dis. 2024 Aug 23;15(8):613. doi: 10.1038/s41419-024-06921-1 (PMC11343739; doi:10.1038/s41419-024-06921-1)

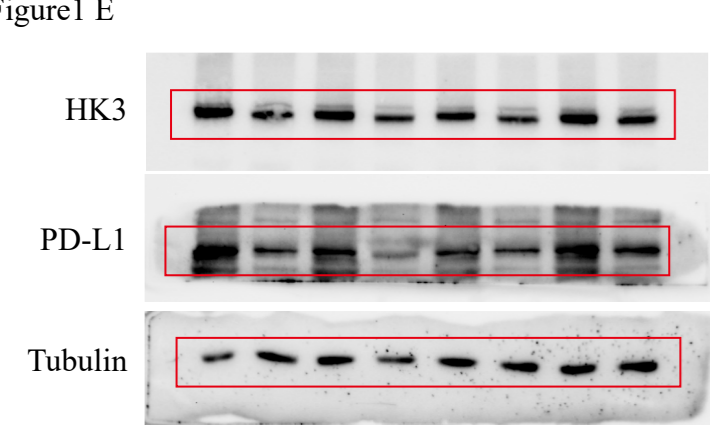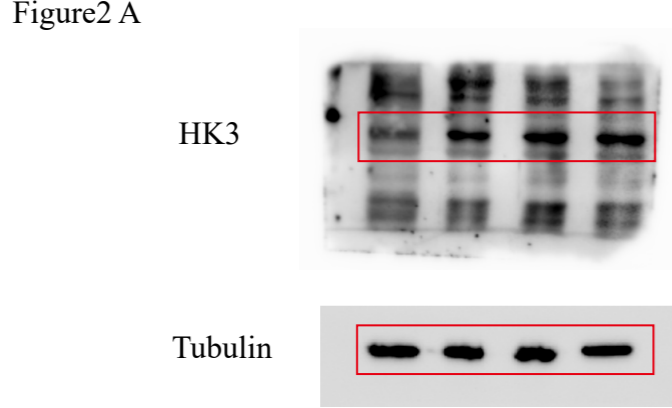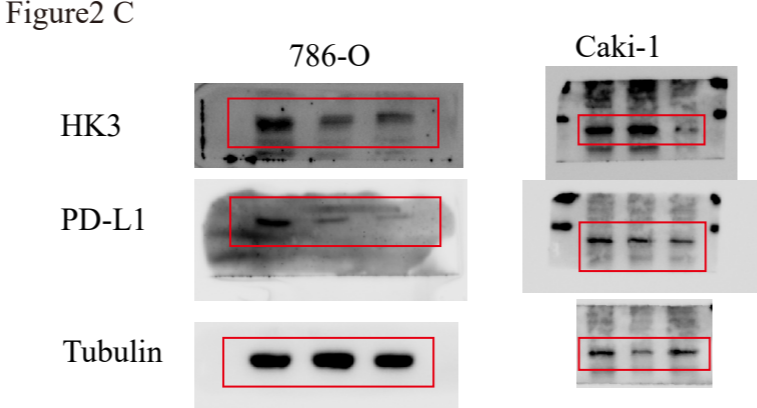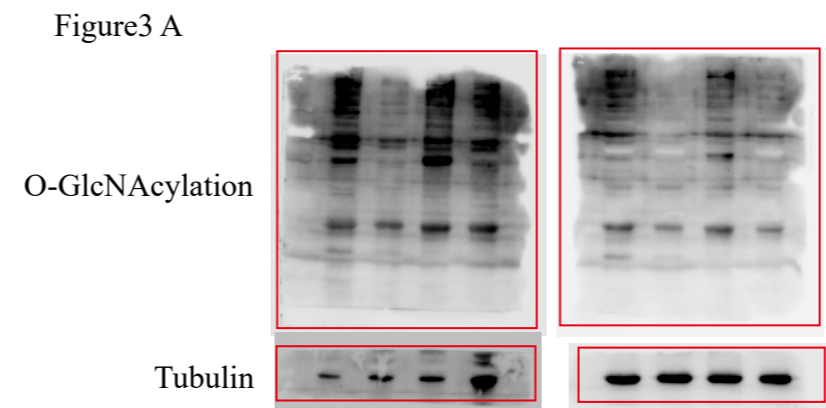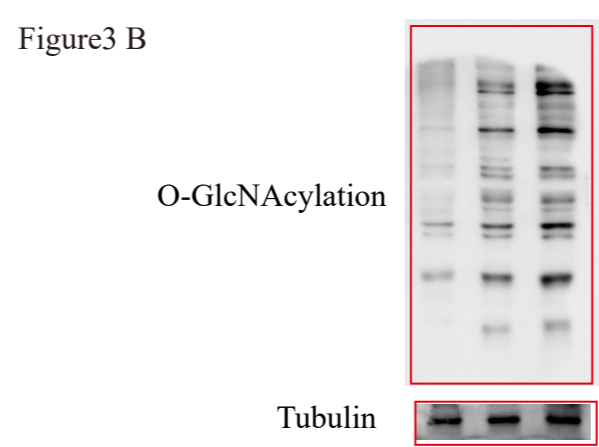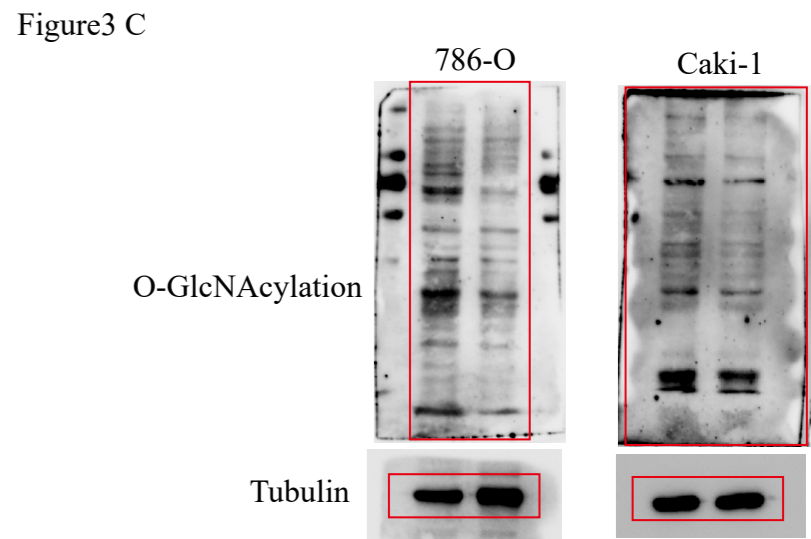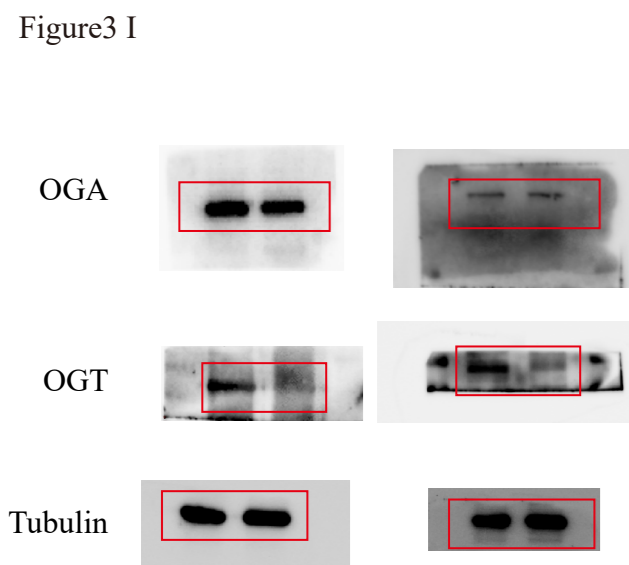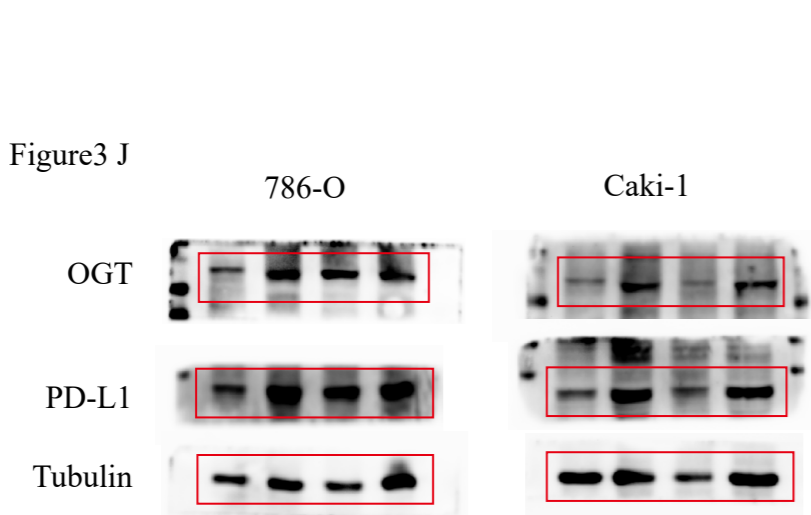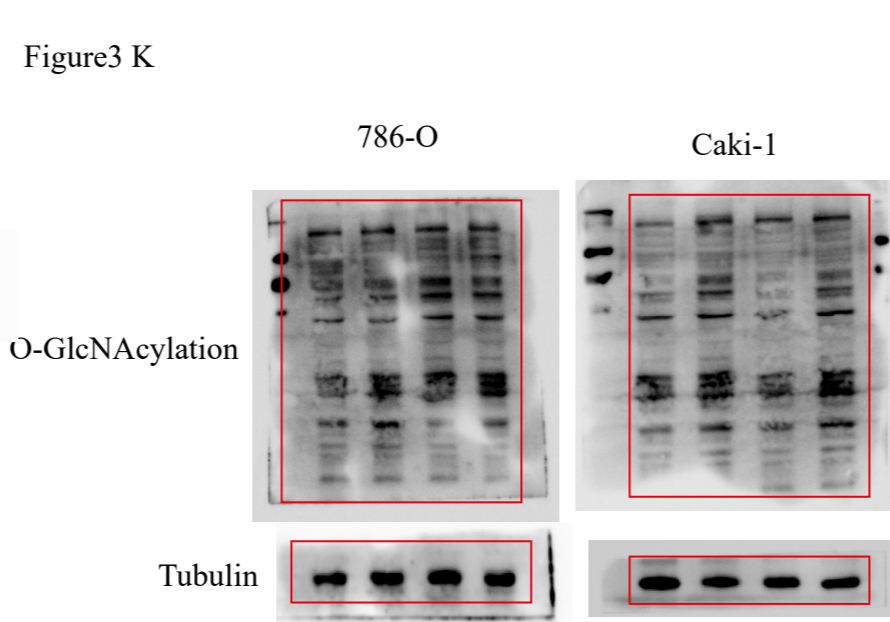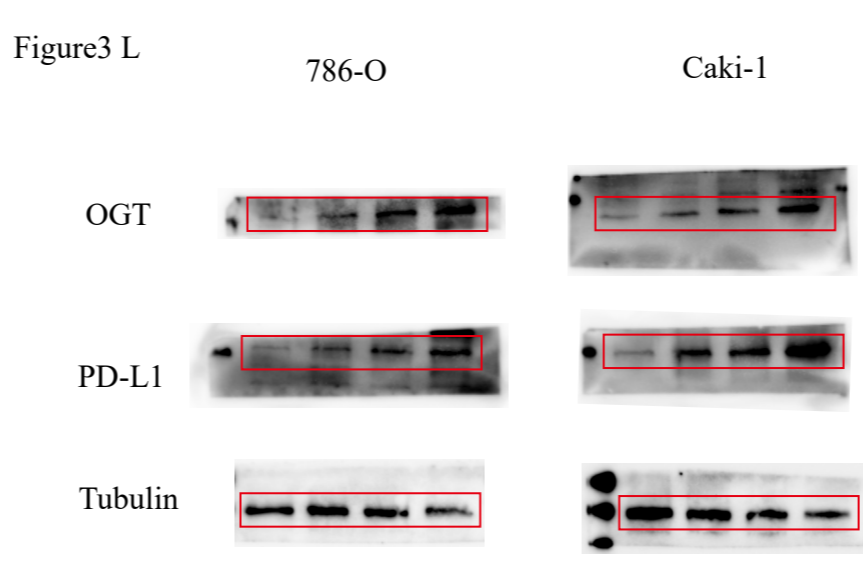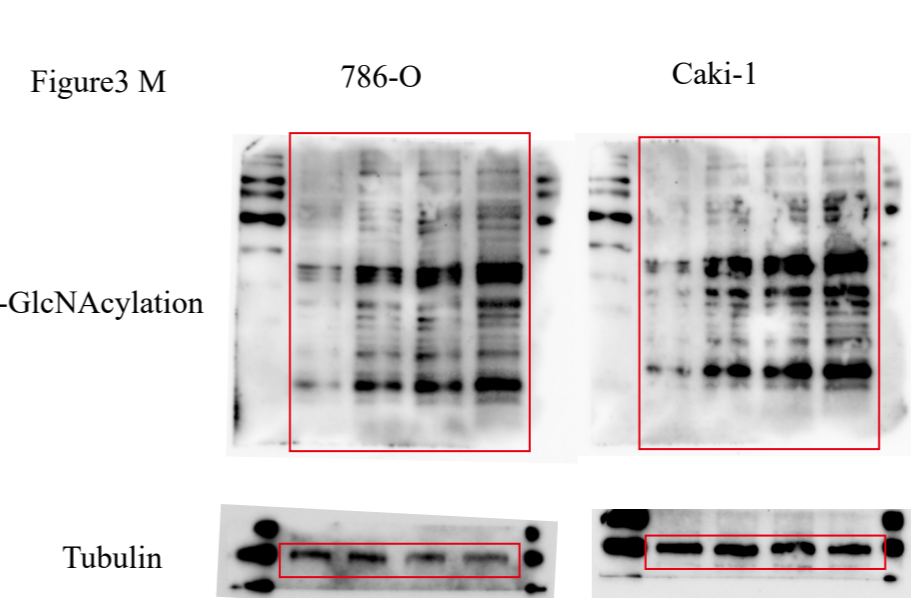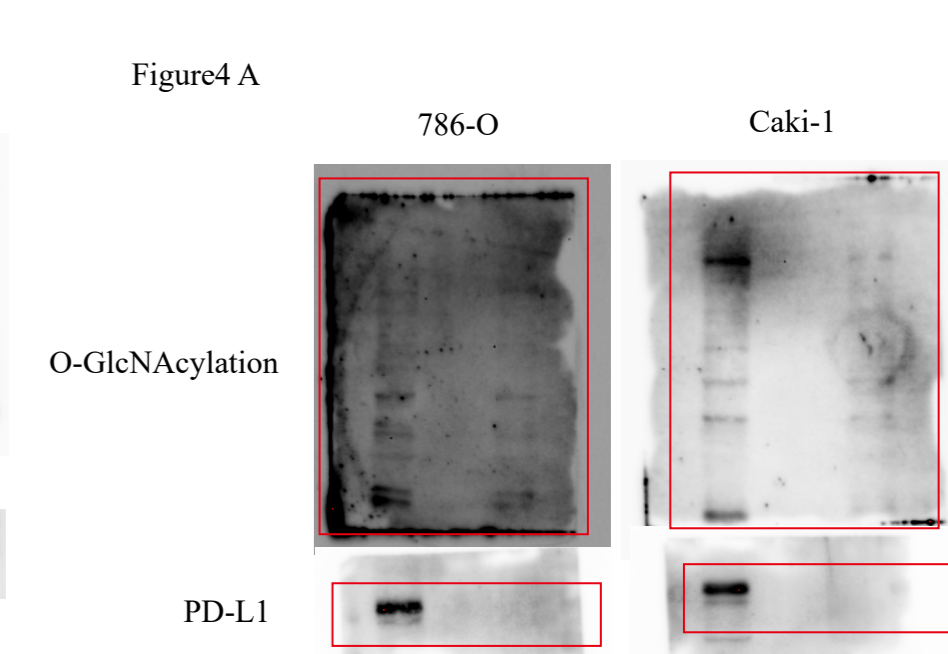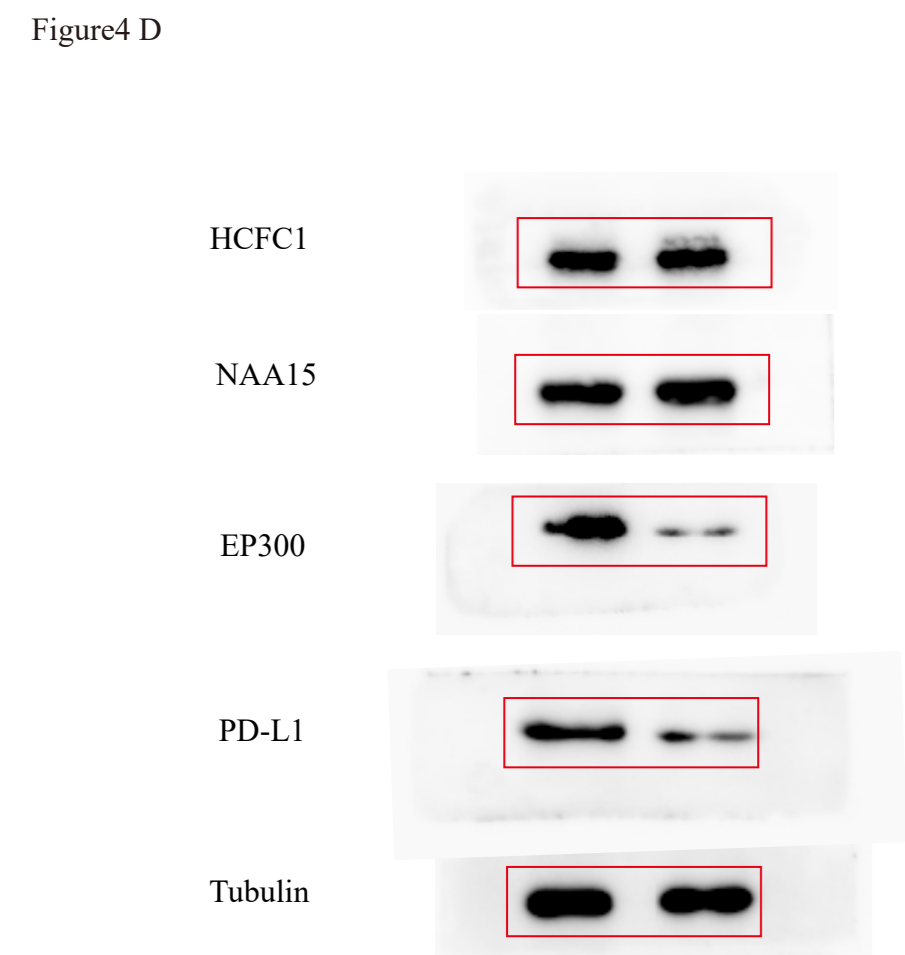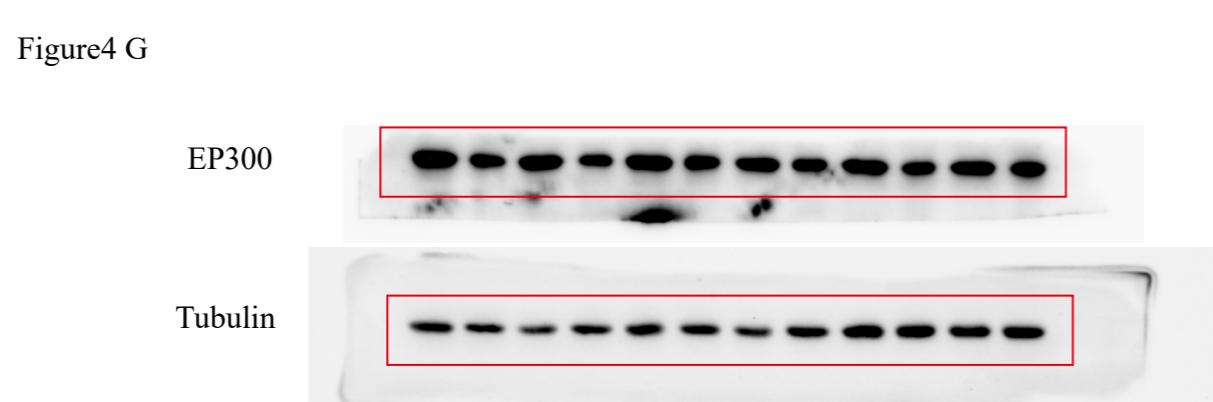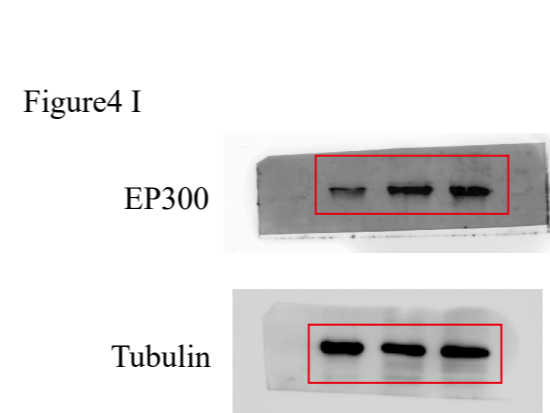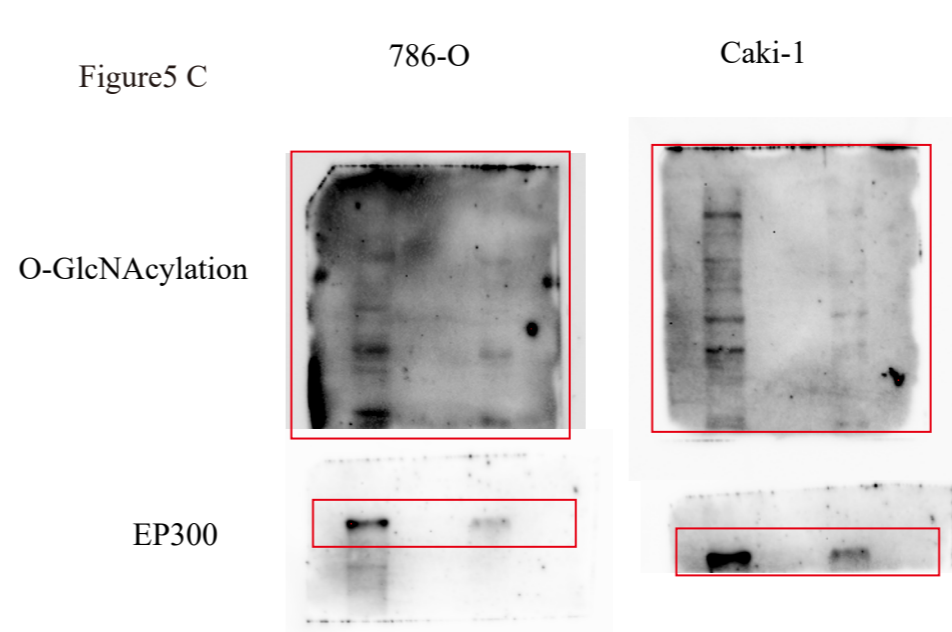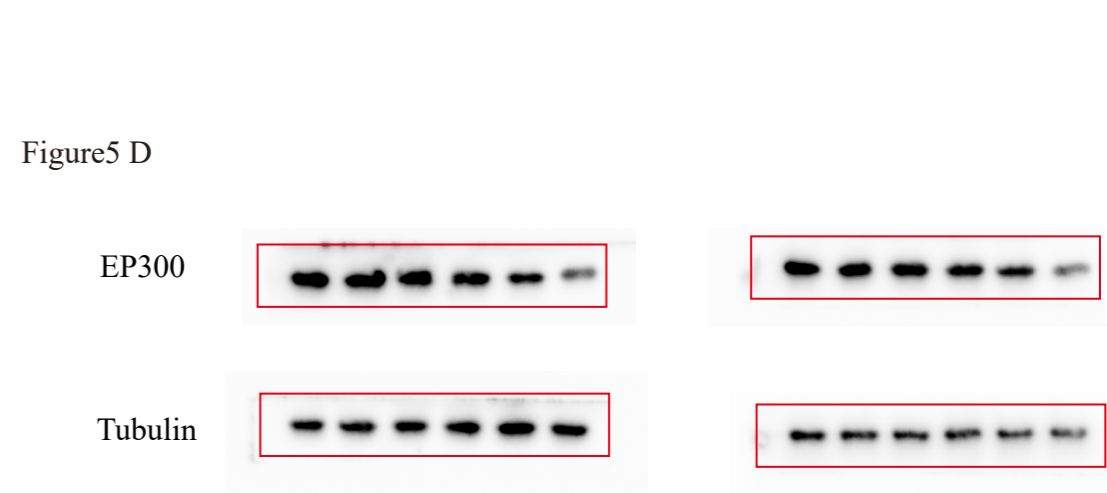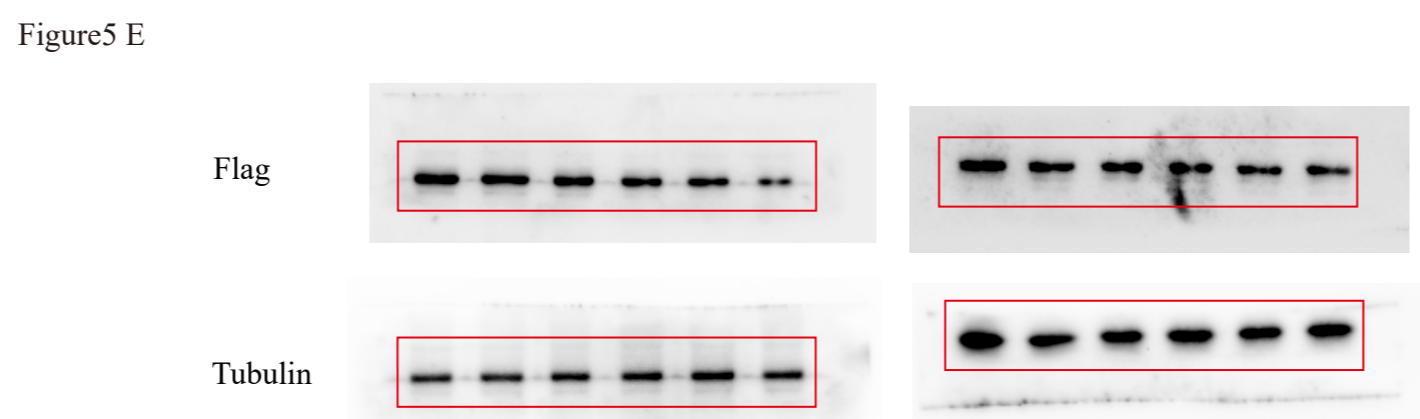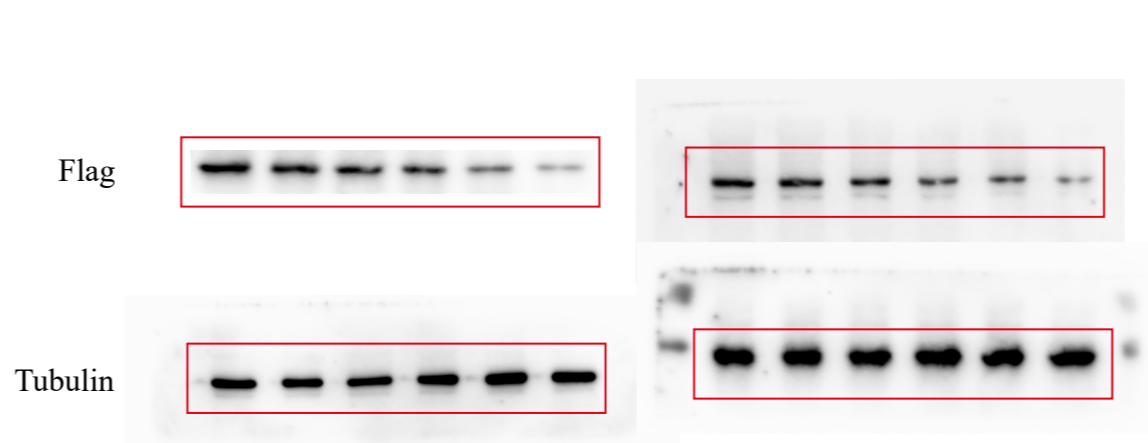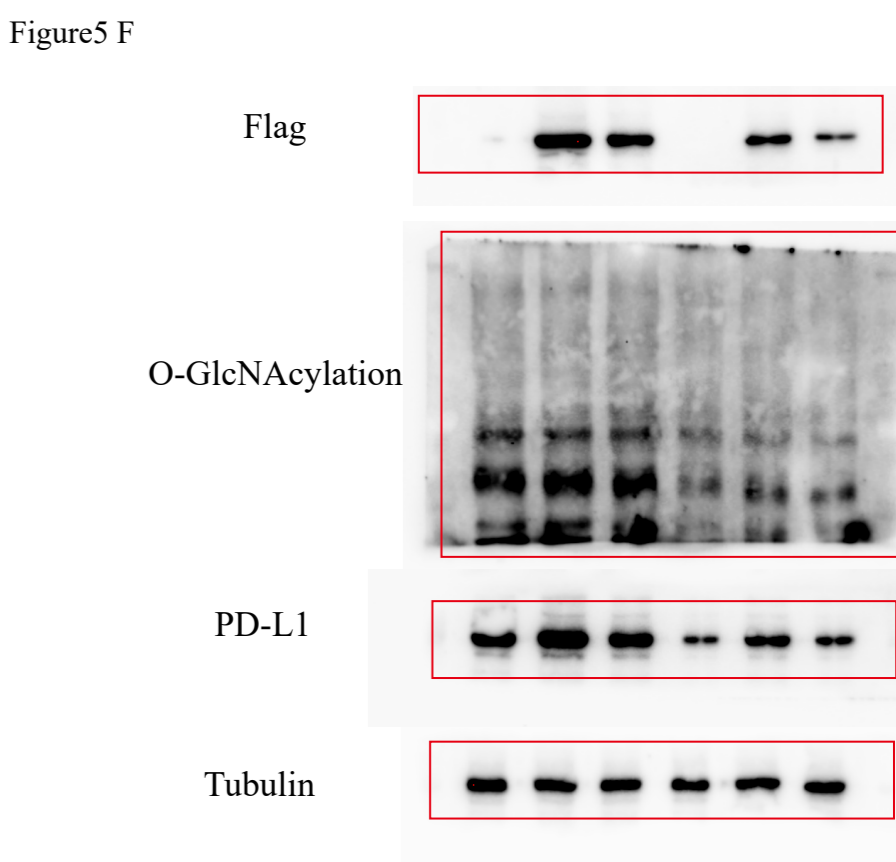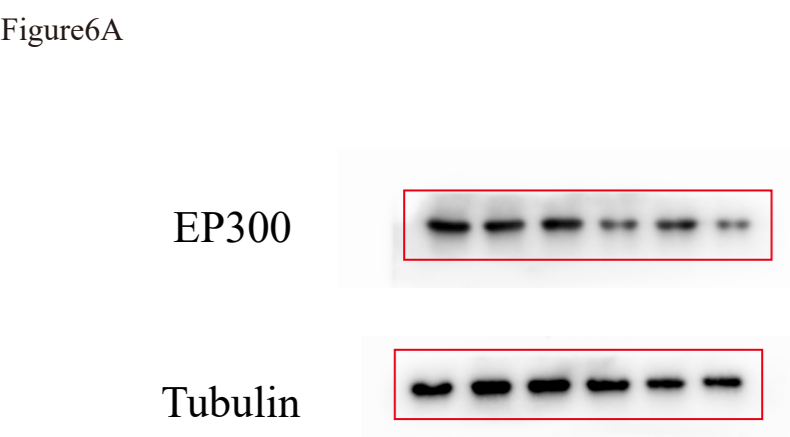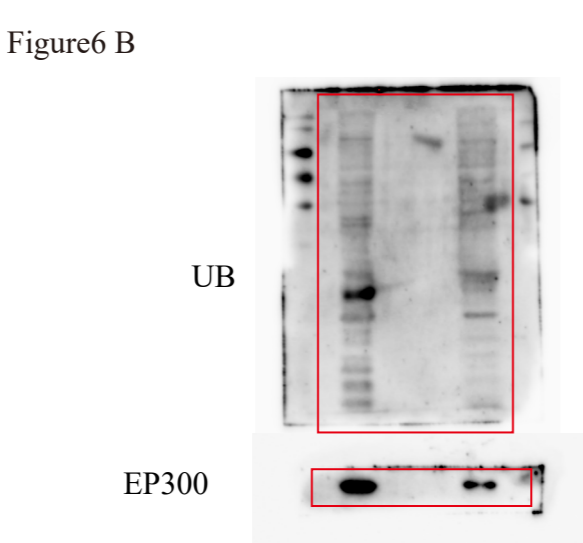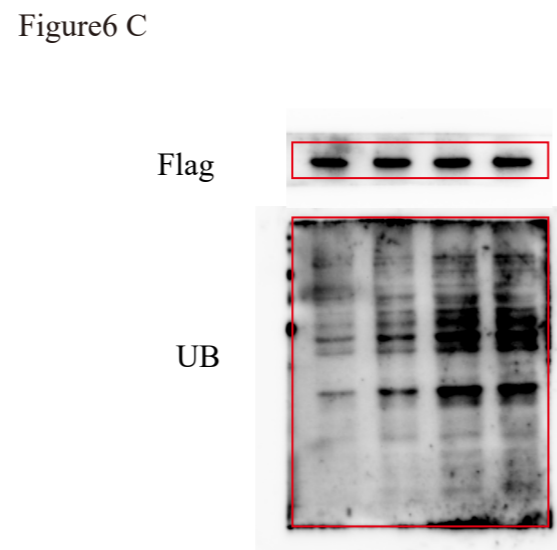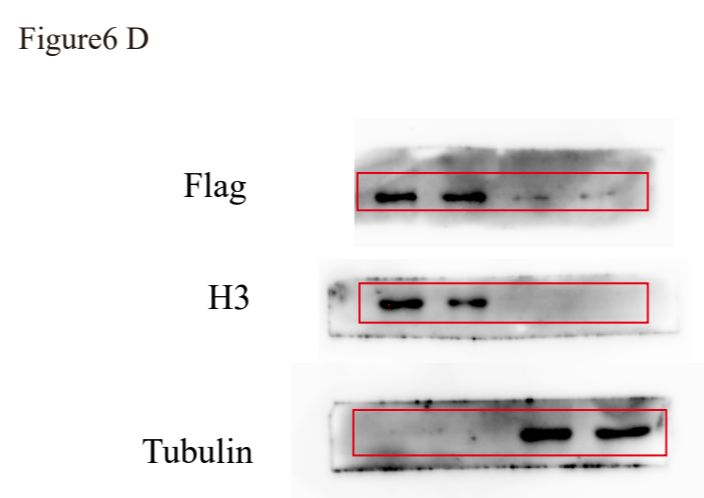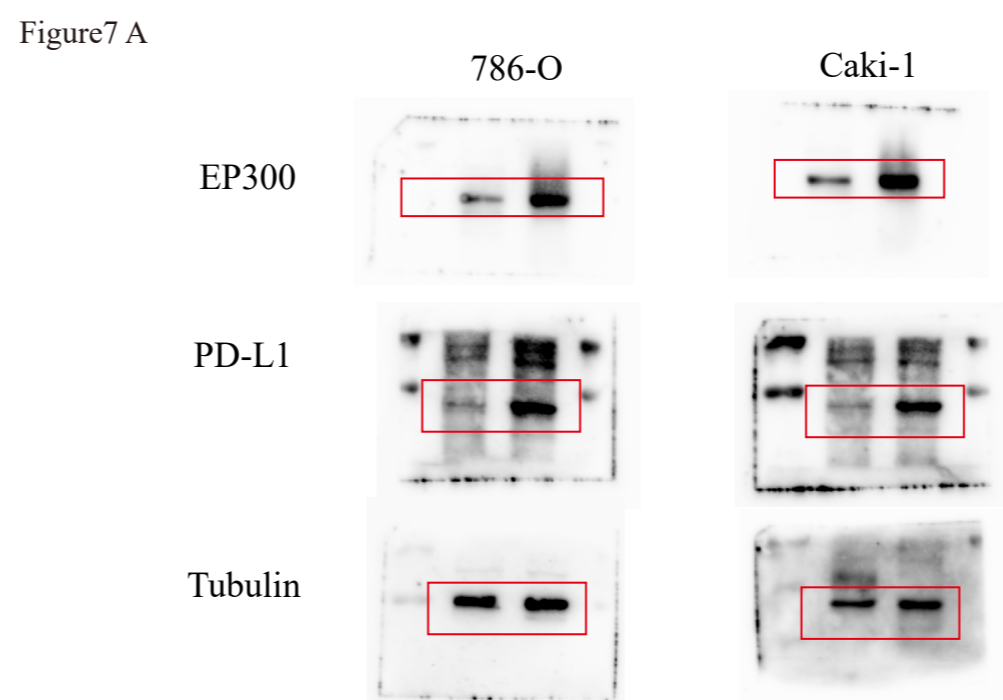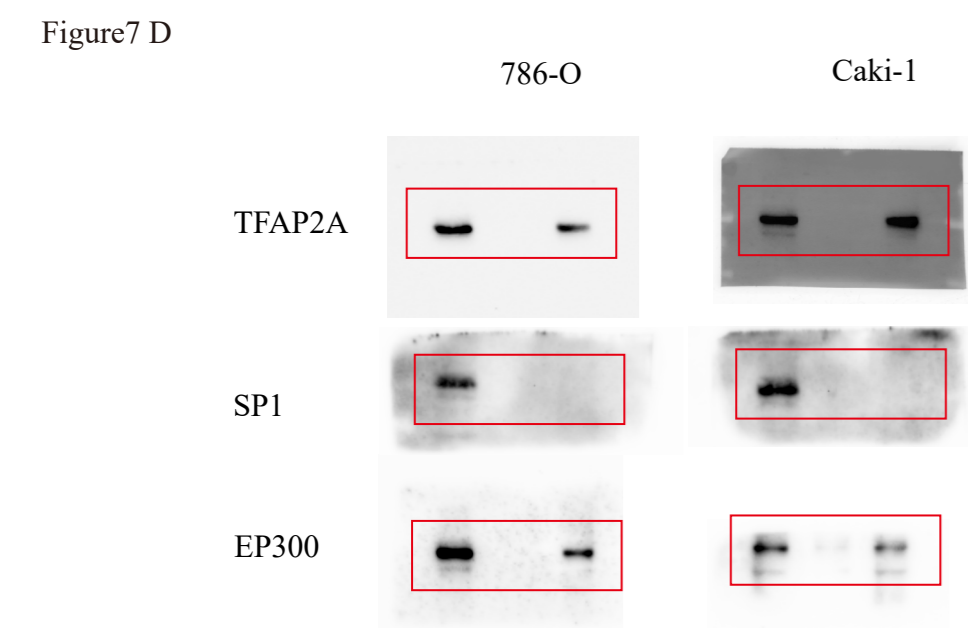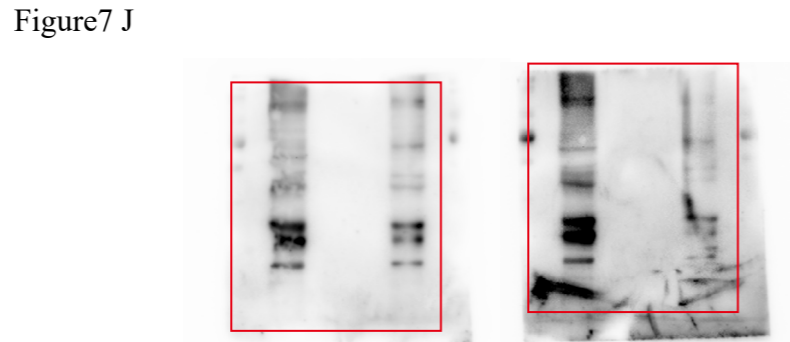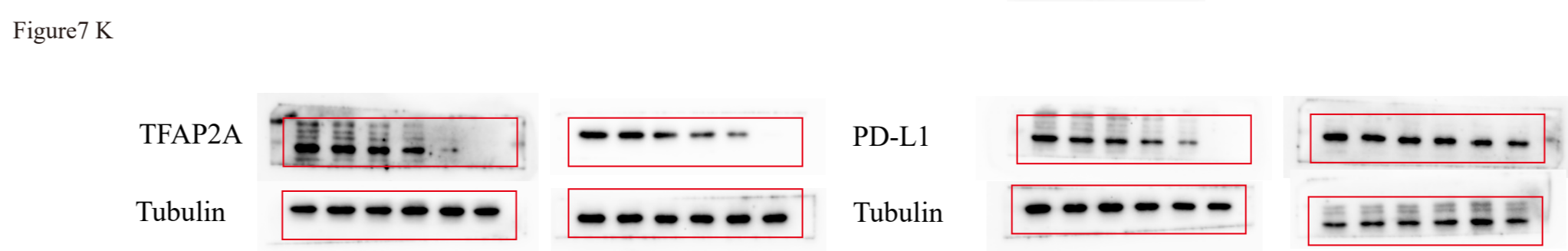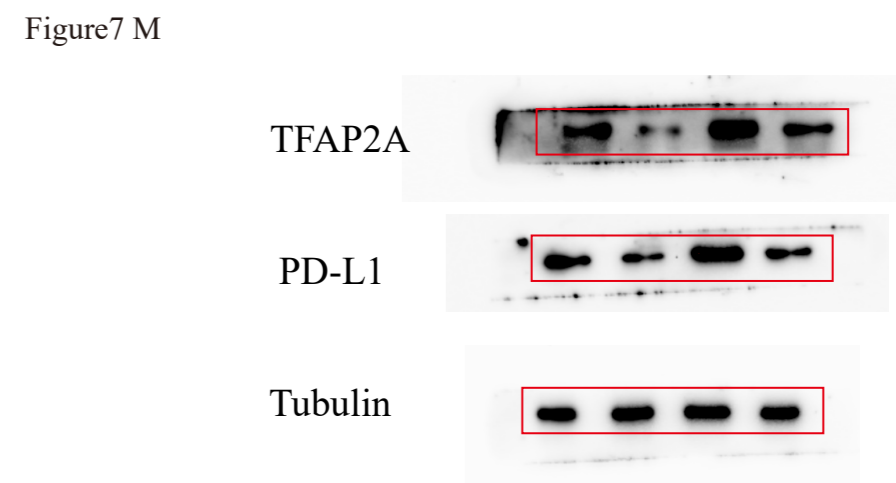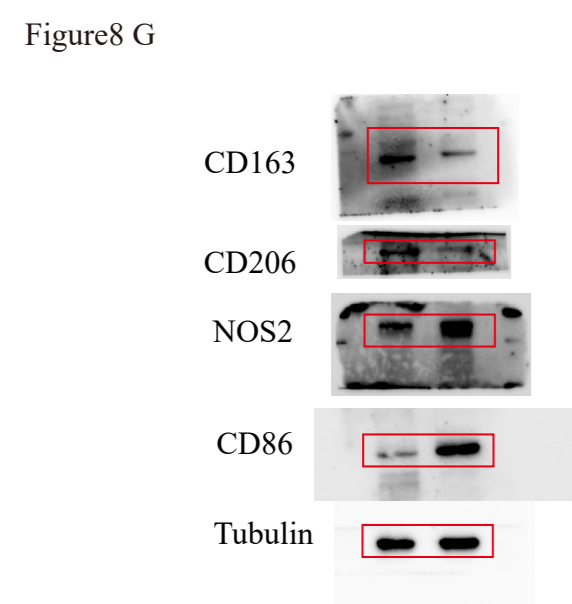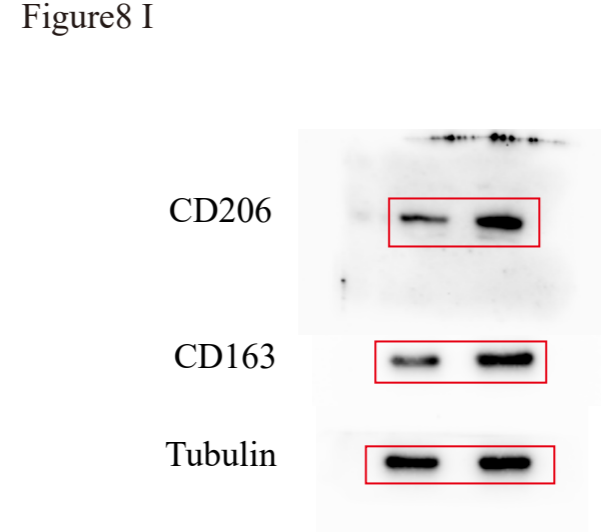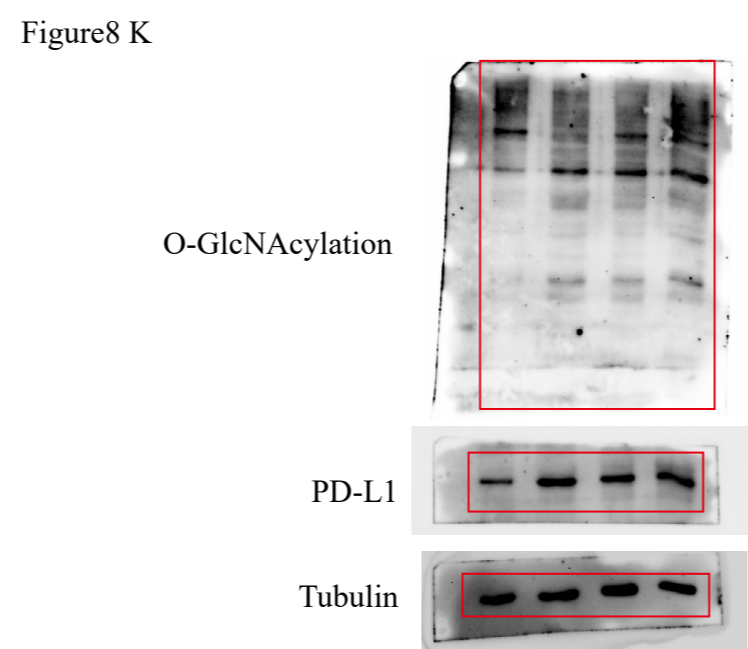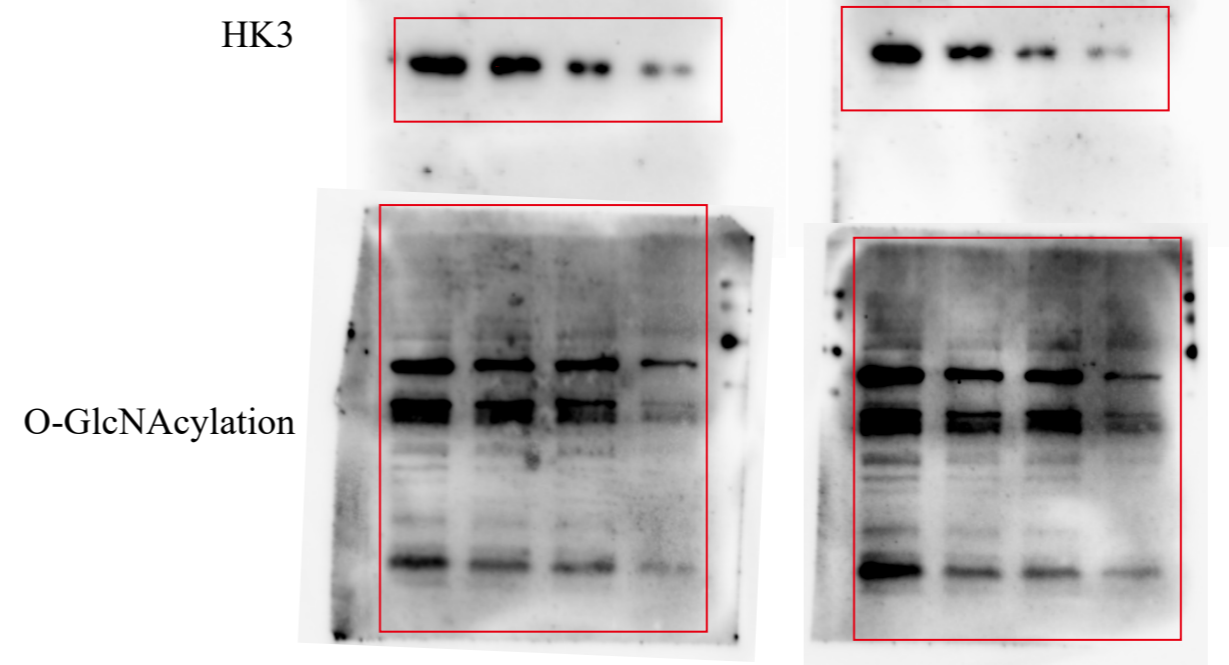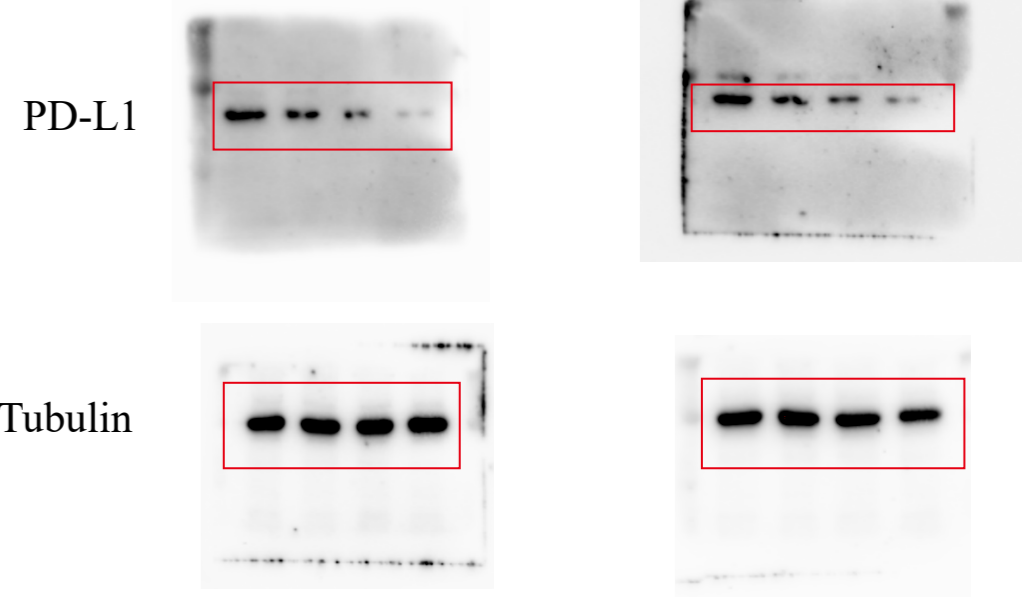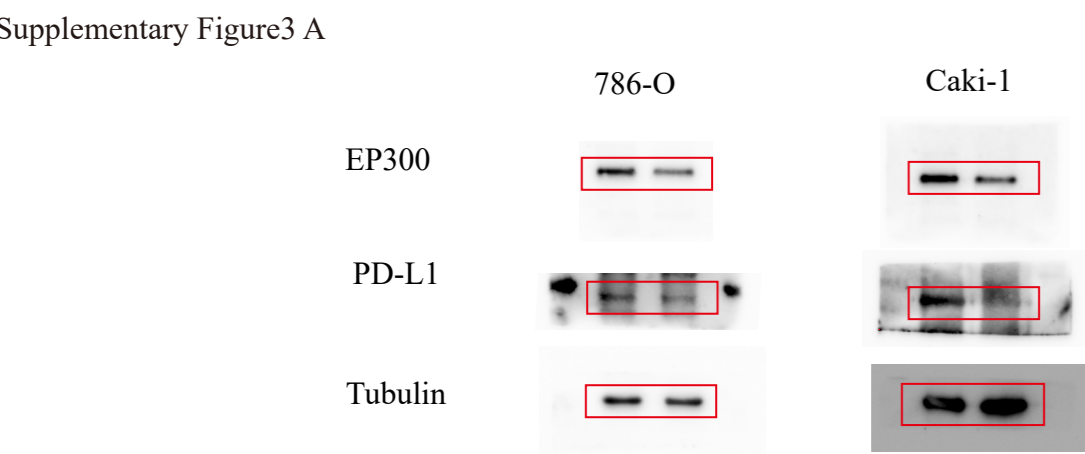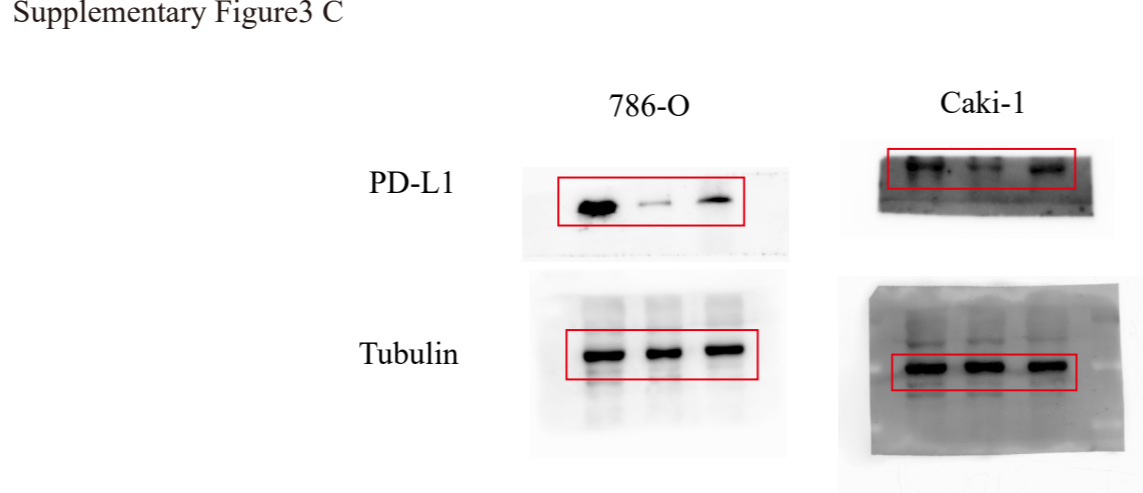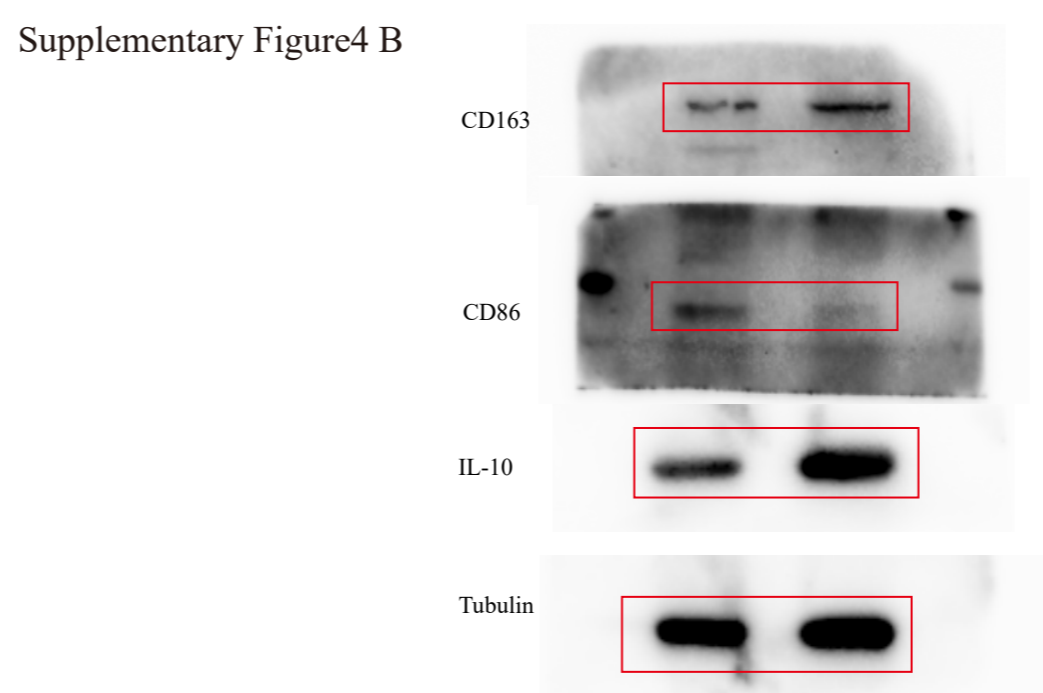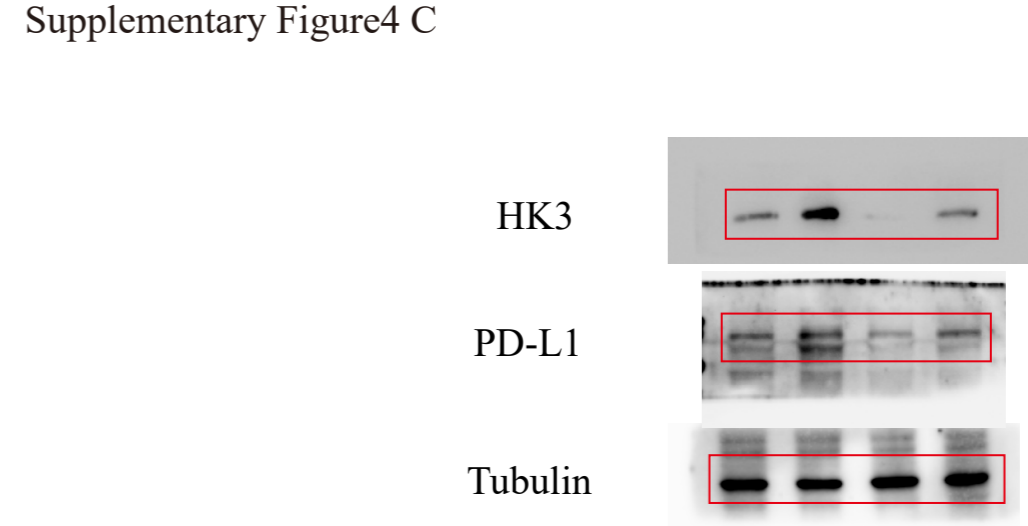

Supplement: Supplementary file 2 — Original western blot [file 41419_2024_6921_MOESM2_ESM.pdf]
